# Supplementary material for: Mitigating the impact of COVID-19 on tuberculosis and HIV services: A cross-sectional survey of 669 health professionals in 64 low and middle-income countries
Source: PLoS One. 2021 Feb 2;16(2):e0244936. doi: 10.1371/journal.pone.0244936 (PMC7853462; doi:10.1371/journal.pone.0244936)
Supplement: S1 File — (ZIP) [file pone.0244936.s001.zip › Arabic.docx]

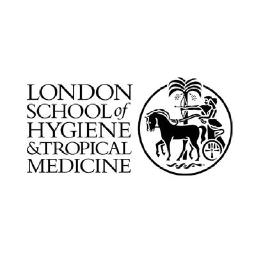


Identifying and mitigating impact of COVID-19 on TB and HIV programmes

تحديد وتخفيف تأثير كوفيد- ١٩ ( على برامج السل وفيروس نقص المناعة البشرية


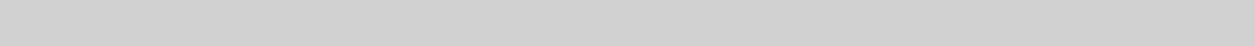


Information / المعلومات


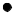
 **We are conducting a short survey to understand ways in which TB and HIV services have been impacted by COVID-19 in low and middle income countries**

**نحن نجري مسحاً قصيراً لفهم الطرق التي تأثرت بها خدمات السل وفيروس نقص المناعة البشرية (HIV) بواسطة كوفيد-١٩ (COVID-19) في البلدان المنخفضة والمتوسطة الدخل**


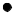
 **The results will help to identify ways to protect and improve TB and HIV services**

**ستساعد النتائج على تحديد سبل حماية وتحسين خدمات السل وفيروس نقص المناعة البشرية (HIV)**


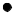
 **This survey is for people who are involved in managing or delivering TB or HIV services (doctors, nurses, policymakers, health facility managers, community groups and researchers). The survey is not intended for patients.**

**هذا المسح مخصص للأشخاص الذين يشاركون في إدارة أو تقديم خدمات السل أو فيروس نقص المناعة البشرية (HIV) (الأطباء والممرضون وواضعي السياسات ومديرو المرافق الصحية ومجموعات المجتمع والباحثون). المسح غير مخصص للمرضى.**


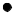
 **You do not have to provide your name or any other details that will allow answers to be traced back to you. All information will be kept completely anonymous.**

**لست مطالب بتقديم اسمك أو أي تفاصيل أخرى تسمح بتتبع الإجابات لك. جميع المعلومات ستبقى مجهولة بالكامل.**


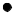
 **Depending on your area of work, you can answer questions about TB (approximately 15 minutes) or HIV (approximately 15 minutes) or both.**

**اعتمادًا على مجال عملك، يمكنك الإجابة عن الأسئلة حول مرض السل (حوالي ١٥ دقيقة) أو فيروس نقص المناعة البشرية (HIV) (حوالي ١٥ دقيقة) أو كليهما.**


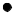
 **Once you start the survey you will need to complete it. You cannot save and come back, so please start the survey when you have enough time (15-30 minutes).**

**بمجرد بدء المسح، ستحتاج إلى إكماله. لا يمكنك حفظه والعودة إليه، لذا يرجى بدء المسح عندما يكون لديك وقت كاف (١٥-٣٠ دقيقة).**


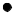
 **Please do not answer this survey more than once.**

**من فضلك لا تجيب على هذا المسح أكثر من مرة.**

**Detailed information about the study and your participation is available to download by clicking** [**here.**](https://docs.google.com/document/d/1L1MrsHnQUj1V72LJV2cYHe7oJlKA_OIAS1gkNWreOIA/edit)

**معلومات تفصيلية عن الدراسة ومشاركتك موجودة للتحميل بالضغط** [**هنا**](https://docs.google.com/document/d/1nAotYpHDFXSoTqi0drU90M-90RbPbwL4/edit)**.**

- 1. **Consent to participate**
- **١. الموافقة على المشاركة**

By clicking the boxes below, I confirm that:

بالنقر على المربعات أدناه ، أؤكد ما يلي:


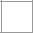


I have agreed to take part in the study

لقد وافقت على المشاركة في الدراسة
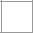


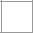


I have seen a copy of the information sheet (available by clicking the link above) that explains my role in this research. I understand its contents and agree to participate in this research.

لقد رأيت نسخة من ورقة المعلومات (متوفرة بالضغط على الرابط أعلاه) التي توضح دوري في هذا البحث. أفهم محتوياته وأوافق على المشاركة في هذا البحث.
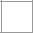


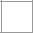


I can withdraw from the survey at any point in time

يمكنني الإنسحاب من المسح في أي وقت
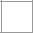


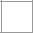


I will not have any financial benefits that result from the commercial development of this research

لن أحصل على أي فوائد مالية ناتجة عن التطوير التجاري لهذا البحث
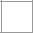


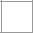


I consent to have the coded data made available for future research by putting it into a data repository

أوافق على أن البيانات المشفرة ستكون متاحة للبحث في المستقبل عن طريق وضعها في مستودع البيانات
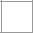


- 2. Thank you for your consent. If you provide text answers, do you agree for us to quote your statements (verbatim) in a report without identifying you?
- ٢. شكراً على موافقتك. إذا قدمتم إجابات نصية ، هل توافق على تمرير إجاباتك (حرفيا) في تقرير دون تحديد هويتك؟


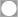
 Yes


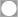
 نعم


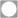
 No


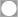
 لا

- 3. What is your age?
- ٣. ما هو عمرك؟
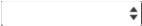

- 4. What is your gender?
- ٤. ما هو جنسك؟


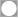
 Female


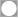
 أنثى


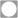
 Male


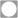
 ذكر


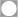
 Prefer not to answer


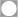
 أفضل عدم الإجابة


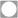
 Prefer to self describe:


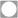
 أفضل وصف الذات:


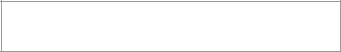


- 5. Which of the following best describes the role you work in?
- ٥. أي مما يلي يصف الدور الذي تعمل فيه بشكل أفضل؟


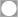
 Nurse providing care to patients


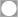
 ممرض(ة) يقدم الرعاية للمرضى


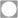
 Doctor providing care to patients


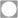
 طبيب(ة) يقدم الرعاية للمرضى


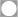
 Community healthcare worker


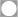
 عامل(ة) الرعاية الصحية المجتمعية


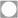
 Other healthcare provider


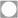
 مقدم(ة) رعاية صحية آخر


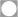
 Manager of healthcare facility or programme


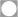
 مدير(ة) منشأة أو برنامج للرعاية الصحية


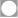
 Researcher


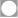
 باحث(ة)


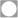
 Other (please specify)


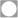
 غير ذلك (يرجى التحديد)


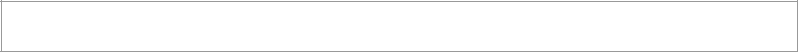


- 6. What type of organisation do you work in?
- ٦. ما نوع المنظمة التي تعمل فيها؟


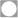
 Public sector healthcare facility


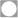
 مرفق الرعاية الصحية في القطاع العام


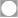
 **Private**, **for-profit** healthcare facility


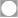
 منشأة رعاية صحية **خاصة ربحية**


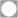
 **Charity**/**non-profit** healthcare facility


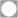
 منشأة رعاية صحية **خيرية / غير ربحية**


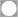
 Government agency


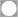
 وكالة حكومية


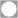
 Domestic non-governmental organisation


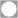
 منظمة غير حكومية محلية


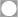
 International non-governmental organisation


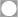
 منظمة دولية غير حكومية


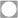
 Funding agency

وكالة التمويل
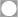


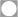
 University or academic body


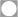
 جامعة أو هيئة أكاديمية


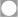
 Other (please specify)


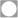
 غير ذلك (يرجى التحديد)


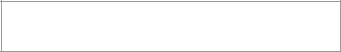


- 7. Which country are you providing information about?
- ٧. ما هي البلد الذي تقدم معلومات عنه؟


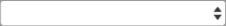


- 8. Please select whether you would like to answer questions on TB, HIV or both
- ٨. يرجى تحديد إذا كنت ترغب في الإجابة عن أسئلة حول السل أو فيروس نقص المناعة البشرية (HIV) أو كليهما


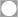
 TB


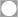
 السل


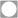
 HIV


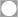
 فيروس نقص المناعة البشرية (HIV)


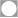
 Both


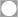
 كليهما

Please answer the 9 short questions on TB. Thank you!

يرجى الإجابة على الأسئلة القصيرة التسعة عن السل. شكراً لك!

**You can select 'prefer not to answer' for any questions you want to skip.**

**يمكنك اختيار "أفضل عدم الإجابة" لأي أسئلة تريد أن تخطيها.**

- 9. Has it been harder for **healthcare providers to come to work** at TB healthcare facilities since COVID-19?
- ٩. هل كان من الصعب على **مقدمي الرعاية الصحية القدوم للعمل** في مرافق الرعاية الصحية لمرض السل منذ كوفبد-١٩ (COVID-19)؟


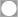
 No - same as before


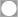
 لا - نفس الشيء من قبل


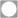
 Yes - it is slightly harder


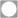
 نعم - إنها أصعب قليلاً


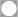
 Yes - it is much harder


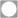
 نعم - إنها أصعب بكثير


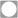
 Yes – it is very difficult or impossible


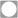
 نعم - إنها صعبة للغاية أو مستحيلة


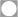
 Don’t know


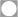
 لا أعلم


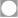
 Prefer not to answer


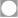
 أفضل عدم الإجابة

- 10. Has it been harder for **TB patients to access TB services** since COVID-19?
- ١٠. هل كان من الصعب على **مرضى السل الوصول إلى خدمات السل** منذ كوفبد-١٩ (COVID-19)؟


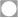
 No - same as before


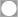
 لا - نفس الشيء من قبل


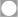
 Yes - it is slightly harder


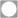
 نعم - إنها أصعب قليلاً


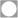
 Yes - it is much harder


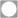
 نعم - إنها أصعب بكثير


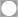
 Yes – it is very difficult or impossible


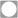
 نعم - إنها صعبة للغاية أو مستحيلة


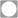
 Don’t know


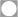
 لا أعلم


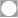
 Prefer not to answer


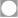
 أفضل عدم الإجابة

- 11. What do you think are the main **concerns or barriers for TB patients** to access healthcare since COVID-19? (select all that apply)
- ١١. برأيك، ما هي **المخاوف أو العوائق الرئيسية أمام مرضى السل** للوصول إلى الرعاية الصحية منذ كوفبد-١٩ (COVID-19)؟

(اختار كل ما ينطبق)


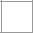


Physical distancing/lockdown rules

قواعد الإبعاد / الإغلاق المادي
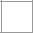


Disruptions to transport

تعطل النقل

Reduced income/access to money to travel

انخفاض الدخل / الحصول على المال للتنقل

Fear of getting infected with COVID-19

الخوف من الإصابة بكوفيد-١٩

Closure of health facilities

إغلاق المنشآت الصحية

Healthcare provider shortages

نقص مقدمي الرعاية الصحية

Longer waiting times

فترات انتظار أطول

Unable to access a face mask

عدم القدرة على الوصول إلى قناع الوجه

There are NO concerns or barriers for TB patients

لا توجد مخاوف أو عوائق لمرضى السل

Prefer not to answer

أفضل عدم الإجابة

Other (please explain below)

غير ذلك (يُرجى التوضيح أدناه)

- 12. Since COVID-19, what **control measures have been implemented by the government** and how has this impacted TB health services? (examples: reduced transport, movement restrictions, etc)
- ١٢. منذ كوفيد-١٩ (COVID-19)، ما هي **تدابير الرقابة التي نفذتها الحكومة** وكيف أثر ذلك على الخدمات الصحية للسل؟ (أمثلة: انخفاض النقل وقيود الحركة وغير ذلك)

- 13. Since COVID-19, are you aware of any changes to the way **TB healthcare facilities are operating**? (select all that apply)
- ١٣. منذ كوفيد-١٩ (COVID-19)، هل أنت على علم بأي تغييرات في طريقة **عمل مرافق الرعاية الصحية لمرض السل**؟ (اختار كل ما ينطبق)

No - same as before

لا - نفس الشيء من قبل

Yes – physical distancing protocols for patients

نعم - بروتوكولات التباعد الجسدي للمرضى

Yes – masks or other protective equipment for healthcare providers

نعم - أقنعة أو معدات حماية أخرى لمقدمي الرعاية الصحية

Prefer not to answer/ don't know

أفضل عدم الإجابة / لا أعرف

Yes - Other, please explain below

نعم - غير ذلك - يُرجى التوضيح أدناه

- 14. Have you experienced shortages of diagnostics or other challenges to provision of routine **diagnostic** **services** for TB since COVID-19?
- ١٤. هل واجهت نقصًا في التشخيص أو تحديات أخرى لتوفير **خدمات التشخيص** الروتينية لمرض السل منذ كوفيد-١٩ (COVID-19)؟

No - same as before

لا - نفس الشيء من قبل

Yes - it is slightly harder to provide diagnostic services

نعم - من الصعب قليلاً تقديم خدمات التشخيص

Yes - it is much harder to provide diagnostic services

نعم - من الصعب تقديم خدمات التشخيص

Yes – it is very difficult or impossible to provide diagnostic services

نعم - من الصعب للغاية أو المستحيل تقديم خدمات التشخيص

Don’t know

لا أعلم

Prefer not to answer

أفضل عدم الإجابة

Please use this space to provide more details about what has caused the change

يُرجى استخدام هذه المساحة لتقديم المزيد من التفاصيل حول سبب التغيير

- 15. Have you experienced shortages of medicines or other challenges to provision of standard **treatment** for TB patients since COVID-19?
- ١٥. هل واجهت نقصًا في الأدوية أو تحديات أخرى لتوفير **العلاج** القياسي لمرضى السل منذ كوفيد-١٩ (COVID-19)؟

No - same as before

لا - نفس الشيء من قبل

Yes - it is slightly harder to provide TB treatment

نعم - من الصعب قليلاً تقديم علاج السل

Yes - it is much harder to provide TB treatment

نعم - من الصعب تقديم علاج السل

Yes – it is very difficult or impossible to provide TB treatment

نعم - من الصعب أو المستحيل تقديم علاج السل

Don’t know

لا أعلم

Prefer not to answer

أفضل عدم الإجابة

Please use this space to provide more details, including challenges with ART for TB patients

يرجى استخدام هذه المساحة لتقديم المزيد من التفاصيل ، بما في ذلك التحديات التي تواجه العلاج بمضادات الفيروسات القهقرية لمرضى السل

- 16. Has it been harder for TB patients to access **non-medical support** such as food supplementation or counselling since COVID-19?
- ١٦. هل كان من الصعب على مرضى السل الوصول إلى **الدعم غير الطبي** مثل المكملات الغذائية أو الاستشارة منذ كوفيد-١٩ (COVID-19)؟

No - same as before

لا - نفس الشيء من قبل

Yes - it is slightly harder

نعم - إنها أصعب قليلاً

Yes - it is much harder

نعم - إنها أصعب بكثير

Yes – it is very difficult or impossible

نعم - إنها صعبة للغاية أو مستحيلة

Not available in my country, region, or facility

غير متوفر في بلدي أو منطقتي أو منشأتي

Don’t know

لا أعلم

Prefer not to answer

أفضل عدم الإجابة

Please use this space to provide more details

يُرجى استخدام هذه المساحة لتقديم المزيد من التفاصيل

1. What do you think can be done (or has already been done) to **minimize or avoid disruptions from** **COVID-19** to TB services?

١٧. برأيك، ما الذي يمكن فعله (أو تم فعله) **لتقليل أو تجنب الاضطرابات من كوفيد-١٩ (COVID-19)** لخدمات السل؟

By clicking the **NEXT** button, you will end this survey. Please check your answers before continuing. Thank you for taking the time to answer this survey!

بالنقر على زر 'التالي' ،ستكمل هذا المسح. الرجاء التحقق من إجاباتك قبل المتابعة. شكراً لك لاعطائك الوقت للإجابة على هذا المسح!

Please answer the 9 short questions on HIV. Thank you!

يرجى الإجابة على الأسئلة القصيرة التسعة عن فيروس نقص المناعة البشرية (HIV). شكراً لك!

**You can select 'prefer not to answer' for any questions you want to skip.**

**يمكنك اختيار "أفضل عدم الإجابة" لأي أسئلة تريد أن تخطيها.**

- 18. Has it been harder for **healthcare providers to come to work** at HIV healthcare facilities since COVID-19?
- ١٨ . هل كان من الصعب على **مقدمي الرعاية الصحية القدوم للعمل** في مرافق الرعاية الصحية لفيروس نقص المناعة البشرية (HIV) منذ كوفبد-١٩ (COVID-19)؟

No - same as before

لا - نفس الشيء من قبل

Yes - it is slightly harder

نعم - إنها أصعب قليلاً

Yes - it is much harder

نعم - إنها أصعب بكثير

Yes – it is very difficult or impossible

نعم - إنها صعبة للغاية أو مستحيلة

Don’t know

لا أعلم

Prefer not to answer

أفضل عدم الإجابة

* 19. Has it been harder for **HIV patients to access HIV services** since COVID-19?

- ١٩. هل كان من الصعب على **مرضى فيروس نقص المناعة البشرية (HIV) الوصول إلى خدمات فيروس نقص المناعة البشرية** **(HIV)** منذ كوفبد-١٩ (COVID-19)؟

No - same as before

لا - نفس الشيء من قبل

Yes - it is slightly harder

نعم - إنها أصعب قليلاً

Yes - it is much harder

نعم - إنها أصعب بكثير

Yes – it is very difficult or impossible

نعم - إنها صعبة للغاية أو مستحيلة

Don’t know

لا أعلم

Prefer not to answer

أفضل عدم الإجابة

* 20. What do you think are the main **concerns or barriers for HIV patients** to access healthcare since COVID-19? (select all that apply)

- ٢٠. برأيك، ما هي **المخاوف أو العوائق الرئيسية أمام مرضى فيروس نقص المناعة البشرية (HIV)** للوصول إلى الرعاية الصحية منذ كوفبد-١٩ (COVID-19)؟

(اختار كل ما ينطبق)

Physical distancing/lockdown rules

قواعد الإبعاد / الإغلاق المادي

Disruptions to transport

تعطل النقل

Reduced income/access to money to travel

انخفاض الدخل / الحصول على المال للتنقل

Fear of getting infected with COVID-19

الخوف من الإصابة بكوفيد-١٩

Closure of health facilities

إغلاق المنشآت الصحية

Healthcare provider shortages

نقص مقدمي الرعاية الصحية

Longer waiting times

فترات انتظار أطول

Unable to access a face mask

عدم القدرة على الوصول إلى قناع الوجه

There are NO concerns or barriers for TB patients

لا توجد مخاوف أو عوائق لمرضى السل

Prefer not to answer

أفضل عدم الإجابة

Other (please explain below)

غير ذلك (يُرجى التوضيح أدناه)

- 21. Since COVID-19, what **control measures have been implemented by the government** and how has this impacted HIV health services? (examples: reduced transport, movement restrictions, etc)
- ٢١. منذ كوفيد-١٩ (COVID-19)، ما هي **تدابير الرقابة التي نفذتها الحكومة** وكيف أثر ذلك على الخدمات الصحية لفيروس نقص المناعة البشرية (HIV)؟ (أمثلة: انخفاض النقل وقيود الحركة وغير ذلك)

* 22. Since COVID-19, are you aware of any changes to the way **HIV healthcare facilities are** **operating**? (select all that apply)

- ٢٢. منذ كوفيد-١٩ (COVID-19)، هل أنت على علم بأي تغييرات في طريقة **عمل مرافق الرعاية الصحية لفيروس نقص المناعة البشرية (HIV)**؟ (اختار كل ما ينطبق)

No - same as before

لا - نفس الشيء من قبل

Yes – physical distancing protocols for patients

نعم - بروتوكولات التباعد الجسدي للمرضى

Yes – masks or other protective equipment for healthcare providers

نعم - أقنعة أو معدات حماية أخرى لمقدمي الرعاية الصحية

Prefer not to answer/ don't know

أفضل عدم الإجابة / لا أعرف

Yes - Other, please explain below

نعم - غير ذلك - يُرجى التوضيح أدناه

- 23. Have you experienced shortages of diagnostics or other challenges to provision of routine **diagnostic** **services** for HIV since COVID-19?
- ٢٣. هل واجهت نقصًا في التشخيص أو تحديات أخرى لتوفير **خدمات التشخيص** الروتينية لفيروس نقص المناعة البشرية (HIV) منذ كوفيد-١٩ (COVID-19)؟

No - same as before

لا - نفس الشيء من قبل

Yes - it is slightly harder to provide diagnostic services

نعم - من الصعب قليلاً تقديم خدمات التشخيص

Yes - it is much harder to provide diagnostic services

نعم - من الصعب تقديم خدمات التشخيص

Yes – it is very difficult or impossible to provide diagnostic services

نعم - من الصعب للغاية أو المستحيل تقديم خدمات التشخيص

Don’t know

لا أعلم

Prefer not to answer

أفضل عدم الإجابة

Please use this space to provide more details about what has caused the change

يُرجى استخدام هذه المساحة لتقديم المزيد من التفاصيل حول سبب التغيير

- 24. Have you experienced shortages of medicines or other challenges to provision of standard **treatment** for HIV patients since COVID-19?
- ٢٤. هل واجهت نقصًا في الأدوية أو تحديات أخرى لتوفير **العلاج** القياسي لمرضى فيروس نقص المناعة البشرية (HIV) منذ كوفيد-١٩ (COVID-19)؟

No - same as before

لا - نفس الشيء من قبل

Yes - it is slightly harder to provide HIV treatment

نعم - من الصعب قليلاً تقديم علاج فيروس نقص المناعة البشرية

Yes - it is much harder to provide HIV treatment

نعم - من الصعب تقديم علاج فيروس نقص المناعة البشرية

Yes – it is very difficult or impossible to provide HIV treatment

نعم - من الصعب أو المستحيل تقديم علاج فيروس نقص المناعة البشرية

Don’t know

لا أعلم

Prefer not to answer

أفضل عدم الإجابة

Please use this space to provide more details

يرجى استخدام هذه المساحة لتقديم المزيد من التفاصيل

- 25. Has it been harder for HIV patients to access **non-medical support** such as food supplementation or counselling since COVID-19?
- ٢٥. هل كان من الصعب على مرضى فيروس نقص المناعة البشرية (HIV) الوصول إلى **الدعم غير الطبي** مثل المكملات الغذائية أو الاستشارة منذ كوفيد-١٩ (COVID-19)؟

No - same as before

لا - نفس الشيء من قبل

Yes - it is slightly harder

نعم - إنها أصعب قليلاً

Yes - it is much harder

نعم - إنها أصعب بكثير

Yes – it is very difficult or impossible

نعم - إنها صعبة للغاية أو مستحيلة

Not available in my country, region, or facility

غير متوفر في بلدي أو منطقتي أو منشأتي

Don’t know

لا أعلم

Prefer not to answer

أفضل عدم الإجابة

Please use this space to provide more details

يُرجى استخدام هذه المساحة لتقديم المزيد من التفاصيل

* 26. What do you think can be done (or has already been done) to **minimize or avoid disruptions from** **COVID-19** to HIV services?

* ٢٦. برأيك، ما الذي يمكن فعله (أو تم فعله) **لتقليل أو تجنب الاضطرابات من كوفيد-١٩ (COVID-19)** لخدمات فيروس نقص المناعة البشرية (HIV)؟

By clicking the **NEXT** button, you will end this survey. Please check your answers before continuing. Thank you for taking the time to answer this survey!

بالنقر على زر **'التالي**' ،ستكمل هذا المسح. الرجاء التحقق من إجاباتك قبل المتابعة. شكراً لك لاعطائك الوقت للإجابة على هذا المسح!

**There will first be 9 short questions about TB, followed by 9 short questions about HIV.**

**سيكون هناك أولاً ٩ أسئلة قصيرة حول السل ، تليها ٩ أسئلة قصيرة حول فيروس نقص المناعة البشرية (HIV).**

**You can select 'prefer not to answer' for any questions you want to skip.**

**يمكنك اختيار "أفضل عدم الإجابة" لأي أسئلة تريد أن تخطيها.**

**Thank you for your time!**

**شكراً لك على وقتك!**

d

- 27. Has it been harder for **healthcare providers to come to work** at TB healthcare facilities since COVID-19?

No - same as before

Yes - it is slightly harder

Yes - it is much harder

Yes – it is very difficult or impossible

Don’t know

Prefer not to answer

- 28. Has it been harder for **TB patients to access TB services** since COVID-19?

No - same as before

Yes - it is slightly harder

Yes - it is much harder

Yes – it is very difficult or impossible

Don’t know

Prefer not to answer

- 29. What do you think are the main **concerns or barriers** for TB patients to access healthcare since COVID-19? (select all that apply)

Physical distancing/lockdown rules

Disruptions to transport

Reduced income/access to money to travel

Fear of getting infected with COVID-19

Closure of health facilities

Healthcare provider shortages

Longer waiting times

Unable to access a face mask

There are NO concerns or barriers for TB patients

Prefer not to answer

Other (please specify)

- 30. Since COVID-19, what **control measures have been implemented by the government** and how has this impacted TB health services? (examples: reduced transport, movement restrictions, etc)

- 31. Since COVID-19, are you aware of any changes to the way **TB healthcare facilities are** **operating**? (select all that apply)

No - same as before

Yes – physical distancing protocols for patients

Yes – masks or other protective equipment for healthcare providers

Prefer not to answer/don't know

Yes- Other. Please explain below

- 32. Have you experienced shortages of diagnostics or other challenges to provision of routine **diagnostic** **services** for TB since COVID-19?

No - same as before

Yes - it is slightly harder to provide diagnostic services

Yes - it is much harder to provide diagnostic services

Yes – it is very difficult or impossible to provide diagnostic services

Don’t know

Prefer not to answer

Please use this space to provide more details about what has caused the change:

- 33. Have you experienced shortages of medicines or other challenges to provision of standard **treatment** for TB patients since COVID-19?

No - same as before

Yes - it is slightly harder to provide TB treatment

Yes - it is much harder to provide TB treatment

Yes – it is very difficult or impossible to provide TB treatment

Don’t know

Prefer not to answer

Please use this space to provide more details

34. Has it been harder for TB patients to access **non-medical support** such as food supplementation or counselling since COVID-19?

No - same as before

Yes - it is slightly harder

Yes - it is much harder

Yes – it is very difficult or impossible

Not available in my country, region, or facility

Don’t know

Prefer not to answer

Please use this space to provide more details

1. What do you think can be done (or has already been done) to **minimize or avoid disruptions from** **COVID-19** to TB services?

Please answer the following 9 questions on HIV

**You can select 'prefer not to answer' for any questions you want to skip.**

- 36. Has it been harder for **healthcare providers to come to work** at HIV healthcare facilities since COVID-19?

No - same as before

Yes - it is slightly harder

Yes - it is much harder

Yes – it is very difficult or impossible

Don’t know

Prefer not to answer

37. Has it been harder for **HIV patients to access HIV services** since COVID-19?

No - same as before

Yes - it is slightly harder

Yes - it is much harder

Yes – it is very difficult or impossible

Don’t know

Prefer not to answer

- 38. What do you think are the main **concerns or barriers for HIV patients** to access healthcare since COVID -19?

Physical distancing/lockdown rules

Disruptions to transport

Reduced income/access to money to travel

Fear of getting infected with COVID-19

Closure of health facilities

Healthcare provider shortages

Longer waiting times

Unable to access a face mask

There are NO concerns or barriers for TB patients

Prefer not to answer/don't know

Other (please specify)

- 39. Since COVID-19, what **control measures have been implemented by the government** and how has this impacted HIV health services? (examples: reduced transport, movement restrictions, etc)

- 40. Since COVID-19, are you aware of any changes to the way **HIV healthcare facilities** are operating? (select all that apply)

No - same as before

Yes – physical distancing protocols for patients

Yes – masks or other protective equipment for healthcare providers

Prefer not to answer/don't know

Yes- Other

- 41. Have you experienced shortages of diagnostics or other challenges to provision of routine **diagnostic** **services** for HIV since COVID-19?

No - same as before

Yes - it is slightly harder to provide diagnostic services

Yes - it is much harder to provide diagnostic services

Yes – it is very difficult or impossible to provide diagnostic services

Don’t know

Prefer not to answer

Please use this space to provide more details about what has caused the change:

- 42. Have you experienced shortages of medicines or other challenges to provision of standard **treatment** for HIV patients since COVID-19?

No - same as before

Yes - it is slightly harder to provide HIV treatment

Yes - it is much harder to provide HIV treatment

Yes – it is very difficult or impossible to provide HIV treatment

Don’t know

Prefer not to answer

Please use this space to provide more details:

- 43. Has it been harder for HIV patients to access **non-medical support** such as food supplementation or counselling since COVID-19?

No - same as before

Yes - it is slightly harder

Yes - it is much harder

Yes – it is very difficult or impossible

Not available in my country, region, or facility

Don’t know

Prefer not to answer

Please use this space to provide more details

1. What do you think can be done (or has already been done) to **minimize or avoid disruptions from** **COVID-19** to HIV services?

By clicking the **DONE** button, you will end this survey. Please check your answers before continuing. Thank you for taking the time to answer this survey!
