# Supplementary material for: Mitigating the impact of COVID-19 on tuberculosis and HIV services: A cross-sectional survey of 669 health professionals in 64 low and middle-income countries
Source: PLoS One. 2021 Feb 2;16(2):e0244936. doi: 10.1371/journal.pone.0244936 (PMC7853462; doi:10.1371/journal.pone.0244936)
Supplement: S1 File — (ZIP) [file pone.0244936.s001.zip › Bahasa_Indonesia_01062020.docx]

**
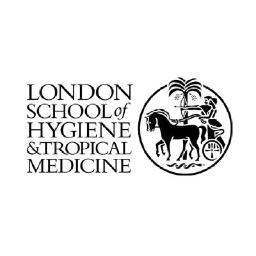
Identifikasi dan Mitigasi dampak pandemi COVID-19 pada program TB dan HIV**


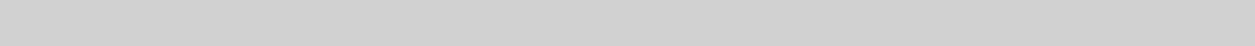


Informasi

**Kami sedang melakukan survei singkat untuk memahami dampak pandemi COVID-19 terhadap program TB dan HIV di negara berkembang.**

- **Hasil studi ini akan membantu merumuskan rekomendasi untuk mendukung implementasi program TB dan HIV**
- **Survei ini ditujukan untuk pihak-pihak yang terlibat dalam mengelola atau mengimplementasikan program TB atau HIV (dokter, perawat, pembuat kebijakan, manajer fasilitas kesehatan, kelompok masyarakat dan peneliti). Survei tidak ditujukan untuk pasien.**
- **Anda tidak perlu memberikan nama Anda atau data diri lainnya. Semua informasi akan disimpan secara anonim.**
- **Sesuai dengan bidang kerja Anda, Anda dapat menjawab pertanyaan tentang TB (sekitar 15 menit) atau HIV (sekitar 15 menit) atau keduanya.**
- **Setelah Anda memulai survei, Anda harus menyelesaikannya. Anda tidak dapat menyimpan jawaban secara sementara dan kemudian mengisi kembali di waktu berbeda, jadi silakan mulai survei ketika Anda memiliki cukup waktu untuk menyelesaikannya. (15-30 menit).**
- **Tolong jangan jawab survei ini lebih dari sekali.**

**Informasi lebih lanjut tentang studi ini dapat diunduh dengan mengklik di sini.**

*** 1. Konfirmasi untuk berpartisipasi**

Dengan mengklik kotak di bawah ini, saya mengonfirmasi bahwa:


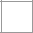


Saya telah setuju untuk terlibat dalam studi ini


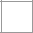


Saya telah melihat salinan lembar informasi (tersedia dengan mengklik tautan di atas) yang menjelaskan peran saya dalam penelitian ini. Saya mengerti isinya dan setuju untuk berpartisipasi dalam studi ini.


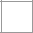


Saya dapat berhenti dari studi ini kapan saja


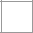
Saya tidak akan memperoleh keuntungan finansial apa pun yang dihasilkan dari pengembangan hasil studi ini secara komersial.


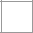
Saya setuju agar jawaban survey ini disimpan dengan data kode dalam repositori LSHTM yang dapat digunakan untuk studi lebih lanjut di masa depan.

* 2. Terima kasih atas persetujuannya. Jika Anda memberikan jawaban teks pada opsi pertanyaan terbuka, apakah Anda setuju kami mengutip pernyataan Anda (kata demi kata) dalam laporan tanpa mengidentifikasi data diri Anda?


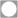
   Iya


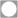
   Tidak

* 3. Berapa umur Anda?


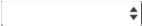


* 4. Apa jenis kelamin Anda?

 
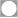
  Perempuan

 
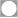
  Pria

 
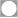
  Memilih tidak menjawab

  
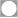
 Memilih untuk mendeskripsikan sendiri:


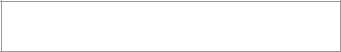


* 5. Manakah dari opsi berikut ini yang paling menggambarkan peran Anda di tempat Anda bekerja?


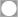
   Perawat yang memberikan perawatan kepada pasien

 
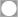
  Dokter yang memberikan perawatan kepada pasien

 
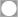
  Petugas kesehatan masyarakat


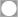


   Penyedia layanan kesehatan lainnya

 
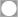
  Manajer fasilitas atau program kesehatan


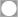


   Peneliti


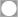


   Lainnya (sebutkan)


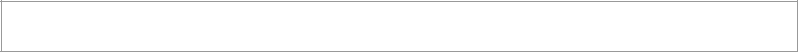


* 6. Apa jenis institusi tempat Anda bekerja?

  
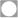
 Fasilitas kesehatan sektor publik


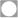


  Fasilitas kesehatan swasta - profit

  
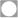
 Fasilitas kesehatan non-profit

  
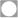
 Lembaga pemerintah

  
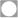
 Lembaga dalam negeri, non-pemerintah

  
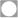
 Lembaga internasional, non-pemerintah


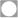
   Lembaga pembiayaan

  
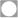
 Universitas atau lembaga akademik


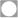


Lainnya (sebutkan)


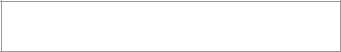


* 7. Di negara mana Anda berasal?


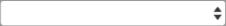


* 8. Silakan pilih apakah Anda ingin menjawab pertanyaan tentang TB, HIV atau keduanya

  
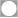
 TB

  
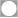
 HIV


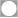


Keduanya

Harap jawab 9 pertanyaan singkat tentang TB. Terima kasih!

**Anda dapat memilih 'memilih untuk tidak menjawab' untuk setiap pertanyaan yang ingin Anda lewati.**

* 9. Sejak pandemi COVID-19, apakah lebih sulit bagi **penyedia layanan kesehatan untuk datang bekerja** ke fasilitas layanan TB?


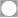
   Tidak - sama seperti sebelumnya


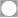
   Ya - sedikit lebih sulit


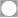
   Ya - jauh lebih sulit


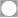
   Ya - sangat sulit atau tidak mungkin


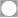
   Tidak tahu

* 10. Apakah lebih sulit bagi **pasien TB untuk mengakses layanan TB** sejak adanya pandemi COVID-19?


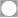
   Tidak - sama seperti sebelumnya


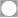
   Ya - sedikit lebih sulit


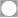
   Ya - jauh lebih sulit


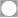
   Ya - sangat sulit atau tidak mungkin


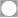
   Tidak tahu


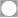


   Memilih untuk tidak menjawab

   * 11. Menurut Anda apa **kekhawatiran atau hambatan utama bagi pasien TB** untuk mengakses layanan kesehatan sejak pandemi COVID-19? (Pilih semua yang berlaku)


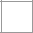


Aturan jaga jarak /*lockdown*


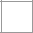
Gangguan transportasi


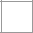


Penurunan pendapatan / biaya untuk bepergian


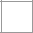


Takut terinfeksi COVID-19


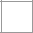
Penutupan fasilitas kesehatan


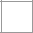
Kurangnya penyedia layanan kesehatan


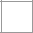


Waktu tunggu yang lebih lama


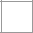


Tidak memiliki masker wajah


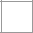


Tidak ada masalah atau hambatan untuk pasien TB


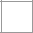


Memilih untuk tidak menjawab


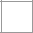


Lainnya (tolong jelaskan di bawah)


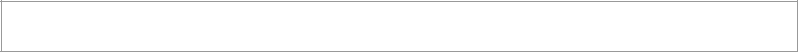


* 12. Sejak pandemi COVID-19, apa **tindakan pengendalian pandemi yang telah dilaksanakan oleh pemerintah** dan bagaimana hal ini berdampak pada layanan kesehatan TB? (contoh: berkurangnya akses transportasi, pembatasan mobilitas, dll)


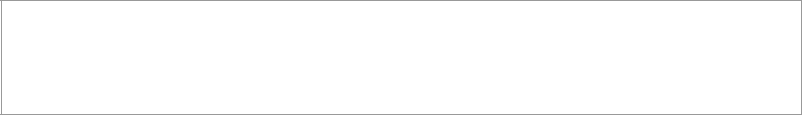


* 13. Sejak pandemi COVID-19, apakah Anda mengetahui adanya perubahan pada implementasi pelayanan TB di **fasilitas kesehatan**? (Pilih semua yang berlaku)


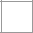
Tidak - sama seperti sebelumnya


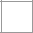
Ya – adanya protokol jaga jarak fisik untuk pasien


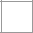


Ya – adanya penyediaan masker atau APD lainnya untuk penyedia layanan kesehatan


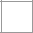


Memilih untuk tidak menjawab / tidak tahu


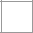
Ya - Lainnya, mohon jelaskan di bawah ini


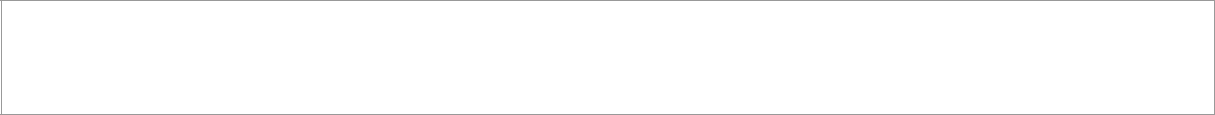


* 14. Pernahkah Anda mengalami kekurangan alat diagnostik atau tantangan lain untuk penyediaan **layanan diagnostik** rutin untuk TB sejak pandemi COVID-19?


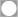
   Tidak - sama seperti sebelumnya


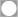
   Ya - sedikit sulit untuk menyediakan layanan diagnostik


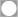
   Ya - jauh lebih sulit untuk menyediakan layanan diagnostik


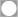
   Ya - sangat sulit atau tidak mungkin untuk menyediakan layanan diagnostik


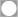
   Tidak tahu


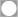
   Memilih untuk tidak menjawab

Silakan gunakan kotak ini untuk memberikan rincian jawaban lebih lanjut tentang penyebab perubahan layanan diagnostik TB


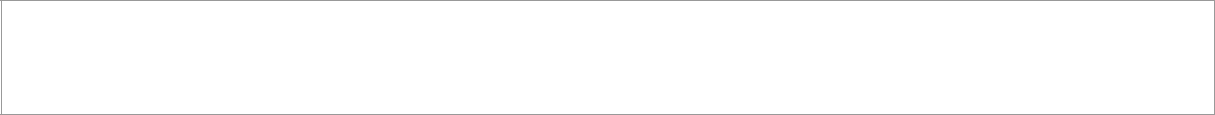


* 15. Pernahkah Anda mengalami kekurangan obat-obatan atau tantangan lain terkait penyediaan **pengobatan** esensial untuk pasien TB sejak pandemi COVID-19?


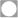
   Tidak - sama seperti sebelumnya


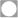
   Ya - sedikit lebih sulit untuk menyediakan pengobatan TB


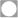
   Ya - jauh lebih sulit untuk menyediakan pengobatan TB


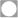
   Ya - sangat sulit atau tidak mungkin untuk menyediakan pengobatan TB


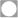
   Tidak tahu


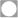
   Memilih untuk tidak menjawab

Silakan gunakan kotak ini untuk memberikan rincian jawaban lebih lanjut


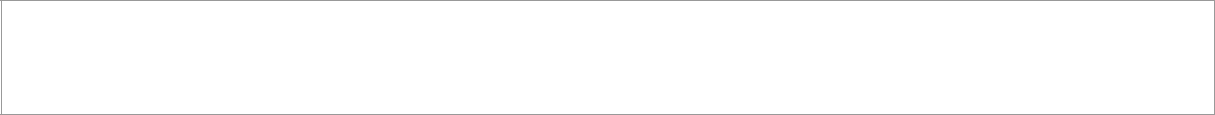


* 16. Apakah lebih sulit bagi pasien TB untuk mengakses **dukungan non-medis** seperti pemberian suplemen makanan atau konseling sejak COVID-19?


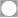
   Tidak - sama seperti sebelumnya


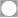
   Ya - sedikit lebih sulit


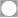
   Ya - jauh lebih sulit


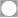
   Ya - sangat sulit atau tidak mungkin


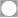
   Tidak tersedia di negara, wilayah, atau fasilitas saya


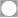
   Tidak tahu


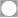
   Memilih untuk tidak menjawab

Silakan gunakan kotak ini untuk memberikan rincian jawaban lebih lanjut


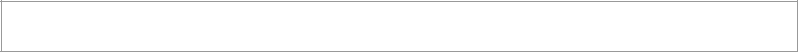


* 17. Menurut Anda, apa yang dapat dilakukan (atau telah dilakukan) untuk **meminimalisasi atau mencegah dampak dari pandemi COVID-19** ke layanan TB?


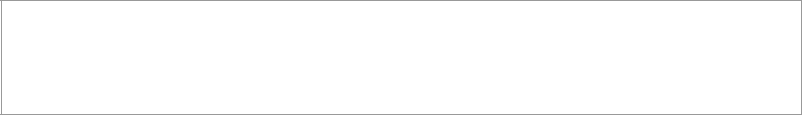


Dengan mengklik tombol **NEXT,** Anda akan mengakhiri survei ini. Silakan periksa jawaban Anda sebelum melanjutkan. Terima kasih telah meluangkan waktu untuk menjawab survei ini!

Harap jawab 9 pertanyaan singkat tentang HIV. Terima kasih!

**Anda dapat memilih 'memilih untuk tidak menjawab' untuk setiap pertanyaan yang ingin Anda lewati.**

* 18. Sejak pandemi COVID-19, apakah lebih sulit bagi **penyedia layanan kesehatan untuk datang bekerja** ke fasilitas layanan HIV?


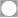
   Tidak - sama seperti sebelumnya


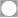
   Ya - sedikit lebih sulit


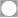
   Ya - jauh lebih sulit


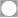
   Ya - sangat sulit atau tidak mungkin


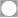
   Tidak tahu


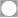
   Memilih untuk tidak menjawab

* 19. Apakah lebih sulit bagi **pasien HIV untuk mengakses layanan HIV** sejak adanya pandemi COVID-19?


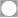
   Tidak - sama seperti sebelumnya


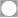
   Ya - sedikit lebih sulit


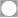
   Ya - jauh lebih sulit


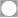
   Ya - sangat sulit atau tidak mungkin

   Tidak tahu

   Memilih untuk tidak menjawab

* 20. Menurut Anda apa **kekhawatiran atau hambatan utama bagi pasien HIV** untuk mengakses layanan kesehatan sejak pandemi COVID-19?

Aturan jaga jarak /*lockdown*

Gangguan transportasi

Penurunan pendapatan / biaya untuk bepergian

Takut terinfeksi COVID-19

Penutupan fasilitas kesehatan

Kurangnya penyedia layanan kesehatan

Waktu tunggu yang lebih lama

Tidak memiliki masker wajah

Tidak ada masalah atau hambatan untuk pasien HIV

Memilih untuk tidak menjawab

Lainnya (tolong jelaskan di bawah)

* 21. Sejak pandemi COVID-19, apa **tindakan pengendalian pandemi yang telah dilaksanakan oleh pemerintah** dan bagaimana hal ini berdampak pada layanan kesehatan HIV? (contoh: berkurangnya akses transportasi, pembatasan mobilitas, dll)

* 22. Sejak pandemi COVID-19, apakah Anda mengetahui adanya perubahan implementasi pelayanan HIV di **fasilitas kesehatan**? (Pilih semua yang berlaku)

Tidak - sama seperti sebelumnya

Ya – adanya protokol jaga jarak fisik untuk pasien

Ya – adanya penyediaan masker atau APD lainnya untuk penyedia layanan kesehatan

Memilih untuk tidak menjawab / tidak tahu

Ya - Lainnya, mohon jelaskan di bawah ini

* 23. Pernahkah Anda mengalami kekurangan alat diagnostik atau tantangan lain untuk penyediaan **layanan diagnostik** rutin untuk HIV sejak pandemi COVID-19?

   Tidak - sama seperti sebelumnya

   Ya - sedikit sulit untuk menyediakan layanan diagnostik

   Ya - jauh lebih sulit untuk menyediakan layanan diagnostik

   Ya - sangat sulit atau tidak mungkin untuk menyediakan layanan diagnostik

   Tidak tahu

   Memilih untuk tidak menjawab

Silakan gunakan kotak ini untuk memberikan rincian jawaban lebih lanjut

* 24. Pernahkah Anda mengalami kekurangan obat-obatan atau tantangan lain terkait penyediaan **pengobatan** esensial untuk pasien HIV sejak pandemi COVID-19?

   Tidak - sama seperti sebelumnya

   Ya - sedikit lebih sulit untuk menyediakan pengobatan HIV

   Ya - jauh lebih sulit untuk menyediakan pengobatan HIV

   Ya - sangat sulit atau tidak mungkin untuk menyediakan pengobatan HIV

   Tidak tahu

   Memilih untuk tidak menjawab

Silakan gunakan kotak ini untuk memberikan rincian jawaban lebih lanjut

* 25. Apakah lebih sulit bagi pasien HIV untuk mengakses **dukungan non-medis** seperti pemberian suplemen makanan atau konseling sejak COVID-19?

   Tidak - sama seperti sebelumnya

   Ya - sedikit lebih sulit

   Ya - jauh lebih sulit

   Ya - sangat sulit atau tidak mungkin

   Tidak tersedia di negara, wilayah, atau fasilitas saya

   Tidak tahu

   Memilih untuk tidak menjawab

Silakan gunakan kotak ini untuk memberikan rincian jawaban lebih lanjut

* 26. Menurut Anda, apa yang dapat dilakukan (atau telah dilakukan) untuk **meminimalisasi atau mencegah dampak dari pandemi COVID-19** ke layanan HIV?

Dengan mengklik tombol **NEXT**, Anda akan mengakhiri survei ini. Silakan periksa jawaban Anda sebelum melanjutkan. Terima kasih telah meluangkan waktu untuk menjawab survei ini!
