# Supplementary material for: Mitigating the impact of COVID-19 on tuberculosis and HIV services: A cross-sectional survey of 669 health professionals in 64 low and middle-income countries
Source: PLoS One. 2021 Feb 2;16(2):e0244936. doi: 10.1371/journal.pone.0244936 (PMC7853462; doi:10.1371/journal.pone.0244936)
Supplement: S1 File — (ZIP) [file pone.0244936.s001.zip › French.docx]

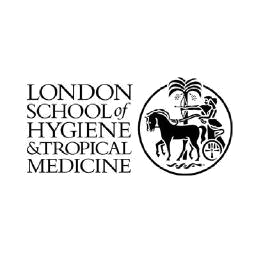


Identification de l’impact de la pandémie de COVID-19 sur les programmes de lutte contre la Tuberculose et le VIH.

Informations

Nous menons une enquête pour comprendre comment les services accueillant des patients tuberculeux (TB) et infectés par le VIH ont été impactés par la pandémie du COVID-19 dans les pays à revenus faibles et moyens.

Les résultats aideront à identifier les moyens de protéger et d'améliorer les services accueillants des patients tuberculeux et infectés par le VIH.

Cette enquête s'adresse aux personnes impliquées dans la gestion ou la prestation de services de lutte contre la tuberculose ou le VIH (médecins, infirmières, responsables, gestionnaires d'établissements de santé, groupes communautaires et chercheurs). L'enquête n'est pas destinée aux patients.

Vous n’avez pas à fournir votre nom ou tout autre détail permettant de vous identifier. Toutes les informations resteront totalement anonymes.

Selon votre domaine de travail, vous pouvez répondre aux questions sur la tuberculose (environ 15 minutes) ou le VIH (environ 15 minutes) ou les deux.

Une fois que vous aurez commencé l'enquête, vous devrez la compléter jusqu’à la fin. Vous ne pouvez pas enregistrer vos réponses puis revenir dessus, veuillez donc commencer l'enquête lorsque vous avez suffisamment de temps pour la compléter (15-30 minutes).

Veuillez ne pas répondre à cette enquête plus d'une fois.

Des informations détaillées sur l'étude et votre participation peuvent être téléchargées en cliquant ici.

* 1. Consentement

En cliquant sur les cases ci-dessous, je confirme que :

J'ai accepté de participer à l'étude.

J'ai lu la fiche d'informations (disponible en cliquant sur le lien ci-dessus) qui explique mon rôle dans cette recherche. Je comprends son contenu et j’accepte de participer à cette recherche.

Je peux me retirer de l'enquête à tout moment.

Je n'aurai aucun avantage financier résultant du développement commercial de cette recherche.

Je consens à ce que les données codées soient disponibles pour de futures recherches en les mettant dans une base de données.

* 2. Merci de votre consentement. Si vous fournissez des réponses textuelles, acceptez-vous que nous citions vos déclarations (textuellement) dans un rapport sans vous identifier ?

   Oui

   Non

* 3. Quel est votre âge ?

* 4. Quel est votre sexe ?

   Féminin

   Masculin

   Je préfère ne pas répondre

   Je m’identifie comme :

* 5. Lequel des énoncés suivants décrit le mieux le rôle dans lequel vous travaillez ?

Infirmière prodiguant des soins aux patients

   Médecin prodiguant des soins aux patients

   Agent de santé communautaire

   Autre professionnel de santé

   Gestionnaire d'établissement ou de programme de santé

   Chercheur

   Autre (veuillez le préciser)

* 6. Dans quel type d'organisation travaillez-vous ?

 Établissement de santé du secteur public

   Établissement de santé privé à but lucratif

   Établissement de santé de bienfaisance / à but non-lucratif

   Organisme gouvernemental

   Organisation non gouvernementale nationale

   Organisation non gouvernementale internationale

   Organisme de financement

   Université ou organisme universitaire

   Autre (veuillez le préciser)

* 7. Sur quel pays fournissez-vous ces informations ?

* 8. Veuillez indiquer si vous souhaitez répondre aux questions sur la tuberculose (TB), le VIH ou les deux

   TB

   VIH

   Les deux

Veuillez, s’il vous plait, répondre aux 9 questions sur la tuberculose. Nous vous en remercions!

Vous pouvez sélectionner « Je préfère ne pas répondre » pour toutes les questions que vous souhaitez ignorer.

* 9. A-t-il été plus difficile pour les professionnels de santé de venir travailler dans les établissements de santé spécialisés dans la lutte contre la tuberculose depuis la pandémie du COVID-19 ?

   Non - comme avant

   Oui - c'est un peu plus difficile

   Oui - c'est beaucoup plus difficile

   Oui - c'est très difficile ou impossible

   Je ne sais pas

   Je préfère ne pas répondre

* 10. A-t-il été plus difficile pour les patients tuberculeux d'accéder aux services de lutte contre la tuberculose depuis la pandémie du COVID-19 ?

   Non - comme avant

   Oui - c'est un peu plus difficile

   Oui - c'est beaucoup plus difficile

   Oui - c'est très difficile ou impossible

   Je ne sais pas

   Je préfère ne pas répondre

* 11. À votre avis, quelles sont les principales préoccupations ou obstacles empêchant les patients tuberculeux d'accéder aux soins depuis la pandémie du COVID-19 ? (Choisir toutes les réponses qui s’y rapportent)

Règles de distanciation sociale / Règles de confinement

Perturbations des transports

Baisse de salaire/ des revenus disponibles pour pouvoir voyager

Peur d'être infecté par le COVID-19

Fermeture des établissements de santé

Pénurie de professionnels de santé

Temps d'attente plus longs

Impossible de se procurer un masque

Il n'y a AUCUNE inquiétude ni aucun obstacle à l’accès aux soins pour les patients tuberculeux

Je préfère ne pas répondre

Autre (veuillez en expliquer ci-dessous)

* 12. Depuis la pandémie du COVID-19 , quelles mesures de contrôle ont été mises en œuvre par le gouvernement et comment ont-elles affecté les services de lutte contre la tuberculose ? (Exemples : transports réduits, restrictions de mouvement, etc.)

* 13. Depuis la pandémie du COVID-19 , êtes-vous au courant de changements dans le fonctionnement des établissements de lutte contre la tuberculose? (Choisir toutes les réponses qui s’y rapportent)

Non - comme avant

Oui - protocoles de distanciation sociale pour les patients

Oui - masques ou autres équipements de protection pour les professionnels de santé

Je préfère ne pas répondre / Je ne sais pas

Oui - Autres, veuillez expliquer ci-dessous :

* 14. Avez-vous connu des pénuries de tests diagnostiques ou d'autres difficultés dans la fourniture de services diagnostiques de routine de la tuberculose depuis la pandémie du COVID-19 ?

   Non - comme avant

   Oui - il est un peu plus difficile de fournir des services diagnostiques

   Oui - il est beaucoup plus difficile de fournir des services diagnostiques

   Oui - il est très difficile ou impossible de fournir des services e diagnostiques

   Je ne sais pas

   Je préfère ne pas répondre

Veuillez décrire plus en détails, ci-dessous la (les) cause(s) du changement :

* 15. Avez-vous connu des pénuries de médicaments ou d'autres difficultés à fournir un traitement standard aux patients tuberculeux depuis la pandémie du COVID-19?

   Non - comme avant

   Oui - il est légèrement plus difficile de fournir un traitement antituberculeux

   Oui - il est beaucoup plus difficile de fournir un traitement antituberculeux

   Oui - il est très difficile ou impossible de fournir un traitement antituberculeux

   Je ne sais pas

   Je préfère ne pas répondre

Veuillez décrire les difficultés rencontrées plus en détails ci-dessous, y compris les défis avec l’ARV (Thérapie antirétrovirale) pour les patients tuberculeux :

* 16. Depuis la pandémie du COVID-19 , a-t-il été plus difficile pour les patients tuberculeux d'accéder à un soutien non médical tel qu'une supplémentation alimentaire ou des conseils ?

Non - comme avant

   Oui - c'est un peu plus difficile

   Oui - c'est beaucoup plus difficile

   Oui - c'est très difficile voire impossible

   Non disponible dans mon pays, ma région ou mon établissement

   Je ne sais pas

   Je préfère ne pas répondre

Veuillez utiliser l’espace ci-dessous pour fournir plus de détails:

17. Selon vous, que peut-on faire (ou a déjà été fait) pour minimiser ou éviter les perturbations dues à la pandémie du COVID-19 sur les services de lutte contre la tuberculose ?

En cliquant sur le bouton SUIVANT, vous terminerez cette enquête. Veuillez vérifier vos réponses avant de continuer. Merci d'avoir pris le temps de répondre à cette enquête !

**Veuillez, s’il vous plait, répondre aux 9 questions sur le VIH. Nous vous en remercions!**

Vous pouvez sélectionner « Je préfère ne pas répondre » pour toutes les questions que vous souhaitez ignorer.

* 18. A-t-il été plus difficile pour les professionnels de santé de venir travailler dans les établissements de santé spécialisés dans la lutte contre le VIH depuis la pandémie du COVID-19 ?

   Non - comme avant

   Oui - c'est un peu plus difficile

   Oui - c'est beaucoup plus difficile

   Oui - c'est très difficile ou impossible

   Je ne sais pas

   Je préfère ne pas répondre

* 19. A-t-il été plus difficile pour les patients tuberculeux d'accéder aux services de lutte contre la VIH depuis la pandémie du COVID-19 ?

   Non - comme avant

   Oui - c'est un peu plus difficile

   Oui - c'est beaucoup plus difficile

   Oui - c'est très difficile ou impossible

   Je ne sais pas

   Je préfère ne pas répondre

* 20. À votre avis, quelles sont les principales préoccupations ou obstacles empêchant les patients VIH d'accéder aux soins depuis la pandémie du COVID-19 ? (Choisir toutes les réponses qui s’y rapportent)

Règles de distanciation sociale / Règles de confinement

Perturbations des transports

Baisse de salaire/ des revenus disponibles pour pouvoir voyager

Peur d'être infecté par le COVID-19

Fermeture des établissements de santé

Pénurie de professionnels de santé

Temps d'attente plus longs

Impossible de se procurer un masque

Il n'y a AUCUNE inquiétude ni aucun obstacle à l’accès aux soins pour les patients VIH

Je préfère ne pas répondre

Autre (veuillez en expliquer ci-dessous)

* 21. Depuis la pandémie du COVID-19 , quelles mesures de contrôle ont été mises en œuvre par le gouvernement et comment ont-elles affecté les services de lutte contre la VIH? (Exemples : transports réduits, restrictions de mouvement, etc.)

* 22. Depuis la pandémie du COVID-19 , êtes-vous au courant de changements dans le fonctionnement des établissements de lutte contre la VIH? (Choisir toutes les réponses qui s’y rapportent)

Non - comme avant

Oui - protocoles de distanciation sociale pour les patients

Oui - masques ou autres équipements de protection pour les professionnels de santé

Je préfère ne pas répondre / Je ne sais pas

Oui - Autres, veuillez expliquer ci-dessous :

* 23. Avez-vous connu des pénuries de tests diagnostiques ou d'autres difficultés dans la fourniture de services diagnostiques de routine de la VIH depuis la pandémie du COVID-19 ?

   Non - comme avant

   Oui - il est un peu plus difficile de fournir des services diagnostiques

   Oui - il est beaucoup plus difficile de fournir des services diagnostiques

   Oui - il est très difficile ou impossible de fournir des services e diagnostiques

   Je ne sais pas

   Je préfère ne pas répondre

Veuillez décrire plus en détails, ci-dessous la (les) cause(s) du changement :

* 24. Avez-vous connu des pénuries de médicaments ou d'autres difficultés à fournir un traitement standard aux patients VIH depuis la pandémie du COVID-19?

   Non - comme avant

   Oui - il est légèrement plus difficile de fournir un traitement VIH

   Oui - il est beaucoup plus difficile de fournir un traitement VIH

   Oui - il est très difficile ou impossible de fournir un traitement VIH

   Je ne sais pas

   Je préfère ne pas répondre

Veuillez décrire les difficultés rencontrées plus en détails ci-dessous, y compris les défis avec l’ARV (Thérapie antirétrovirale) pour les patients VIH:

* 25. Depuis la pandémie du COVID-19 , a-t-il été plus difficile pour les patients VIH d'accéder à un soutien non médical tel qu'une supplémentation alimentaire ou des conseils ?

Non - comme avant

   Oui - c'est un peu plus difficile

   Oui - c'est beaucoup plus difficile

   Oui - c'est très difficile voire impossible

   Non disponible dans mon pays, ma région ou mon établissement

   Je ne sais pas

   Je préfère ne pas répondre

Veuillez utiliser l’espace ci-dessous pour fournir plus de détails:

26. Selon vous, que peut-on faire (ou a déjà été fait) pour minimiser ou éviter les perturbations dues à la pandémie du COVID-19 sur les services de lutte contre le VIH ?

En cliquant sur le bouton SUIVANT, vous terminerez cette enquête. Veuillez vérifier vos réponses avant de continuer. Merci d'avoir pris le temps de répondre à cette enquête !
