# Supplementary material for: Mitigating the impact of COVID-19 on tuberculosis and HIV services: A cross-sectional survey of 669 health professionals in 64 low and middle-income countries
Source: PLoS One. 2021 Feb 2;16(2):e0244936. doi: 10.1371/journal.pone.0244936 (PMC7853462; doi:10.1371/journal.pone.0244936)
Supplement: S1 File — (ZIP) [file pone.0244936.s001.zip › Portuguese.docx]

- **Estamos a executar um pequeno questionário para entender como os serviços de Tuberculose e HIV foram afetados pelo COVID-19 em países de baixa e média renda.**
- **Os resultados ajudarão a identificar maneiras de proteger e melhorar os serviços de Tuberculose e HIV.**
- **Esta pesquisa é para pessoas envolvidas na gestão e fornecimento de serviços de Tuberculose ou HIV (médicos, enfermeiros, responsáveis de elaboração de políticas, gestores de unidades de saúde, grupos comunitários e pesquisadores). A pesquisa não se destina a pacientes.**
- **Você não precisa fornecer o seu nome ou outros detalhes que permitam rastrear as respostas. Todas as informações serão mantidas completamente anónimas.**
- **Dependendo da sua área de trabalho, você pode responder a perguntas sobre Tuberculose (aproximadamente 15 minutos) ou HIV (aproximadamente 15 minutos) ou ambos.**
- **Depois de iniciar o questionário, é necessário terminá-lo. Você não poderá salvar e voltar; portanto, inicie o questionário quando tiver tempo suficiente (15 a 30 minutos).**
- **Por favor, não responda a este questionário mais de uma vez.**

Informações detalhadas sobre o estudo e a sua participação estão disponíveis para download clicando aqui.

* 1. Consentimento em participar

Ao clicar nas opções abaixo, confirmo que:

Eu concordei em participar no estudo.

Vi uma cópia da folha de informações (disponível clicando no link acima) que explica o meu papel nesta pesquisa. Entendo o seu conteúdo e concordo em participar nesta pesquisa.

Posso sair do questionário a qualquer momento.

Não terei nenhum benefício financeiro resultante do desenvolvimento comercial desta pesquisa.

Autorizo que os dados codificados se disponibilizem para pesquisas futuras, colocando-os num arquivo de dados.

* 2. Obrigado pelo seu consentimento. Se você fornecer respostas em texto, concorda em que citemos as suas declarações (literalmente) num relatório sem identificá-lo?

   Sim

   Não

* 3. Qual é a sua idade?

* 4. Qual é o seu sexo?

    Femenino

    Masculino

    Prefiro não responder

    Prefiro me auto-descrever:

* 5. Qual das seguintes alternativas melhor descreve a função que você desempenha?

    Enfermeira que presta cuidados aos pacientes

    Médico que presta cuidados aos pacientes

    Trabalhador comunitário de saúde

    Outro profissional de saúde

    Gerente duma instalação ou programa de saúde

    Investigador

    Outro (por favor, especifique)

* 6. Em que tipo de organização você trabalha?

   Instituição de saúde do setor público

   Instituição de saúde privada com fins lucrativos

   Instituição de assistência médica sem finalidades lucrativas

   Agência do governo

   Organização não governamental nacional

   Organização não governamental internacional

   Agência financiadora

   Universidade ou órgão académico

   Outro (por favor, especifique)

* 7. De que país você está a fornecer informações?

* 8. Por favor, selecione se você gostaria de responder perguntas sobre Tuberculose, HIV ou ambos

   Tuberculose

   HIV

   Ambos

Responda às 9 perguntas breves sobre Tuberculose. Obrigado!

Você pode selecionar 'prefiro não responder' para todas as perguntas que você quer ignorar.

* 9. Tem sido mais difícil para os profissionais de saúde trabalharem nas unidades de saúde dedicadas à Tuberculose, desde o COVID-19?

   Não - o mesmo de antes

   Sim - é um pouco mais difícil

   Sim - é muito mais difícil

   Sim - é muito difícil ou impossível

   Não sabe

   Prefiro não responder

* 10. Tem sido mais difícil para os pacientes com Tuberculose ter acesso aos serviços de Tuberculose desde o COVID-19?

   Não - o mesmo de antes

   Sim - é um pouco mais difícil

   Sim - é muito mais difícil

   Sim - é muito difícil ou impossível

   Não sabe

   Prefiro não responder

* 11. Quais são as principais preocupações ou barreiras para os pacientes com Tuberculose acederem aos cuidados de saúde desde o COVID-19? (selecione tudo que se aplique)

Regras de distanciamento / confinamento

Interrupções no transporte

Redução dos rendimentos/ acesso reduzido ao dinheiro para viajar

Medo de se infectar com COVID-19

Encerramento de unidades de saúde

Escassez de profissionais de saúde

Tempos de espera mais longos

Não foi possivel ter acesso a uma máscara facial

Não há preocupações ou barreiras para pacientes com Tuberculose

Prefiro não responder

Outro (explique abaixo)

* 12. Desde o COVID-19, que medidas de controle foram implementadas pelo governo e como isso afetou os serviços de saúde da Tuberculose? (exemplos: transporte reduzido, restrições de movimento, etc.)

* 13. Desde o COVID-19, você está ciente de alguma alteração na maneira como os serviços de saúde da Tuberculose estão a funcionar? (seleccione tudo que se aplique)

Não - o mesmo de antes

Sim - protocolos de distanciamento entre pacientes

Sim - máscaras ou outro equipamento de proteção para profissionais de saúde

Prefiro não responder / não sei

Sim - Outro, explique abaixo

* 14. Você já enfrentou escassez de ferramentas diagnósticas ou outros desafios à prestação de serviços de diagnóstico de rotina para Tuberculose desde o COVID-19?

   Não - o mesmo de antes

   Sim - é um pouco mais difícil fornecer serviços de diagnóstico

   Sim - é muito mais difícil fornecer serviços de diagnóstico

   Sim - é muito difícil ou impossível fornecer serviços de diagnóstico

   Não sabe

   Prefiro não responder

Usa este espaço para fornecer mais detalhes sobre o que causou a mudança

* 15. Você já sentiu escassez de medicamentos ou outros desafios à provisão do tratamento padrão para pacientes com Tuberculose desde o COVID-19?

   Não - o mesmo de antes

   Sim - é um pouco mais difícil fornecer tratamento para Tuberculose

   Sim - é muito mais difícil fornecer tratamento para Tuberculose

   Sim - é muito difícil ou impossível fornecer tratamento para Tuberculose

   Não sei

   Prefiro não responder

Use este espaço para fornecer mais detalhes, incluindo desafios com os anti-retrovirais para os pacientes com Tuberculose

* 16. Foi mais difícil para os pacientes com Tuberculose acederem ao apoio não médico, como os suplementos alimentares ou o aconselhamento desde o COVID-19?

   Não - o mesmo de antes

   Sim - é um pouco mais difícil

   Sim - é muito mais difícil

   Sim - é muito difícil ou impossível

   Não disponível no meu país, região ou instalação

   Não sei

   Prefiro não responder

Usa este espaço para fornecer mais detalhes

17. O que você acha que pode ser feito (ou que já foi feito) para minimizar ou evitar interrupções do COVID-19 nos serviços de Tuberculose?

Ao clicar no botão SEGUE você vai terminar este questionário. Por favor, verifique as suas respostas antes de continuar. Obrigado por ter respondido aõ questionário!

Responda às 9 perguntas breves sobre VIH. Obrigado!

Você pode selecionar 'prefiro não responder' para todas as perguntas que você quer ignorar.

* 18.. Tem sido mais difícil para os profissionais de saúde trabalharem nas unidades de saúde dedicadas à VIH desde o COVID-19?

   Não - o mesmo de antes

   Sim - é um pouco mais difícil

   Sim - é muito mais difícil

   Sim - é muito difícil ou impossível

   Não sabe

   Prefiro não responder

*19. Tem sido mais difícil para os pacientes com VIH ter acesso aos serviços de VIH desde o COVID-19?

   Não - o mesmo de antes

   Sim - é um pouco mais difícil

   Sim - é muito mais difícil

   Sim - é muito difícil ou impossível

   Não sabe

   Prefiro não responder

20 Quais são as principais preocupações ou barreiras para os pacientes com VIH acederem aos cuidados de saúde desde o COVID-19? (selecione tudo que se aplique)

Regras de distanciamento / confinamento

Interrupções no transporte

Redução dos rendimentos/ acesso reduzido ao dinheiro para viajar

Medo de se infectar com COVID-19

Encerramento de unidades de saúde

Escassez de profissionais de saúde

Tempos de espera mais longos

Não foi possivel ter acesso a uma máscara facial

Não há preocupações ou barreiras para pacientes com VIH

Prefiro não responder

Outro (explique abaixo)

21. Desde o COVID-19, que medidas de controle foram implementadas pelo governo e como isso afetou os serviços de saúde da VIH? (exemplos: transporte reduzido, restrições de movimento, etc.)

22 Desde o COVID-19, você está ciente de alguma alteração na maneira como os serviços de saúde da VIH estão a funcionar? (seleccione tudo que se aplique)

Não - o mesmo de antes

Sim - protocolos de distanciamento entre pacientes

Sim - máscaras ou outro equipamento de proteção para profissionais de saúde

Prefiro não responder / não sei

Sim - Outro, explique abaixo

23 Você já enfrentou escassez de ferramentas diagnósticas ou outros desafios à prestação de serviços de diagnóstico de rotina para VIH desde o COVID-19?

   Não - o mesmo de antes

   Sim - é um pouco mais difícil fornecer serviços de diagnóstico

   Sim - é muito mais difícil fornecer serviços de diagnóstico

   Sim - é muito difícil ou impossível fornecer serviços de diagnóstico

   Não sabe

   Prefiro não responder

Usa este espaço para fornecer mais detalhes sobre o que causou a mudança

24 Você já sentiu escassez de medicamentos ou outros desafios à provisão do tratamento padrão para pacientes com VIH desde o COVID-19?

   Não - o mesmo de antes

   Sim - é um pouco mais difícil fornecer tratamento para VIH

   Sim - é muito mais difícil fornecer tratamento para VIH

   Sim - é muito difícil ou impossível fornecer tratamento para VIH

   Não sei

   Prefiro não responder

Use este espaço para fornecer mais detalhes, incluindo desafios com os anti-retrovirais para os pacientes com VIH

25 Foi mais difícil para os pacientes com VIH acederem ao apoio não médico, como os suplementos alimentares ou o aconselhamento desde o COVID-19?

   Não - o mesmo de antes

   Sim - é um pouco mais difícil

   Sim - é muito mais difícil

   Sim - é muito difícil ou impossível

   Não disponível no meu país, região ou instalação

   Não sei

   Prefiro não responder

Usa este espaço para fornecer mais detalhes

26 O que você acha que pode ser feito (ou que já foi feito) para minimizar ou evitar interrupções do COVID-19 nos serviços de VIH?
