# Supplementary material for: Mitigating the impact of COVID-19 on tuberculosis and HIV services: A cross-sectional survey of 669 health professionals in 64 low and middle-income countries
Source: PLoS One. 2021 Feb 2;16(2):e0244936. doi: 10.1371/journal.pone.0244936 (PMC7853462; doi:10.1371/journal.pone.0244936)
Supplement: S1 File — (ZIP) [file pone.0244936.s001.zip › SHONA .docx]

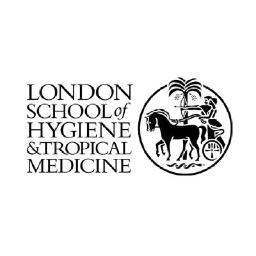


Identifying and mitigating impact of COVID-19 on TB and HIV programmes

Kuziva nekuderedza kukanganisa kweCOVID-19 kuzvirongwa zveTB neHIV


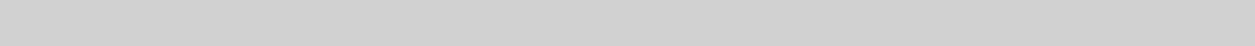


Information

Ruzivo

- **We are conducting a short survey to understand ways in which TB and HIV services have been impacted by COVID-19 in low and middle income countries**
- **Tirikuita ongororo pfupi kuti tinzwisise nzira dzekuti mabasa eTB neHIV akakanganiswa zvakadii munyika dzakasaririra neidzo dziripakati nepakati kubvira patanga COVID-19.**
- **The results will help to identify ways to protect and improve TB and HIV services**
- **Zvichabuda zvichabatsira kuwana nzira dzekudzivirira kana kuwandudza mabasa ezveTB neHIV**
- **This survey is for people who are involved in managing or delivering TB or HIV services (doctors, nurses, policymakers, health facility managers, community groups and researchers). The survey is not intended for patients.**
- **Mibvunzo iyi ndeye vanhu avo vanotungamirira kana kuita mabasa ezveTB neHIV (vanachiremba, vanamukoti, vanonyora mitemo, vanotungamirira mabasa epazvipatara, vemapato emunharaunda nevaongorori). Mibvunzo iyi haisi yevarwere.**
- **You do not have to provide your name or any other details that will allow answers to be traced back to you. All information will be kept completely anonymous.**
- **Hamusikutarisirwa kunyora zita renyu kana chii zvacho chinoita kuti muzivikanwe. Zvese zvamuchanyora zvichange zvisingaite kuti pasava neanoziva kuti zvanyorwa nani.**
- **Depending on your area of work, you can answer questions about TB (approximately 15 minutes) or HIV (approximately 15 minutes) or both.**
- **Zvichienderana nebasa renyu, munogona kupindura mibvunzo nezveTB (zvenguva inogona kuita maminitsi gumi nemashanu (15)) kana nezveHIV (zvenguva inogona kuita maminitsi gumi nemashanu (15))**
- **Once you start the survey you will need to complete it. You cannot save and come back, so please start the survey when you have enough time (15-30 minutes).**
- **Kana mangotanga kupindura mibvunzo iyi munofanira kuipedza. Hamukwanisa kumbochengeta muchizodzokazve, ndapota tangai kupindura mibvunzo apo pamunenge muine nguva yakakwana (maminitsi 15-30).**
- **Please do not answer this survey more than once.**
- **Ndapota musapinduro mibvunzo iyi kanodarika kamwechete.**

**Detailed information about the study and your participation is available to download by clicking** [**here.**](https://docs.google.com/document/d/1L1MrsHnQUj1V72LJV2cYHe7oJlKA_OIAS1gkNWreOIA/edit)

**Zvose zvamungada kuziva nezvekuva kwenyu muchirongwa ichi munozviwana** [**pano**](https://docs.google.com/document/d/1L1MrsHnQUj1V72LJV2cYHe7oJlKA_OIAS1gkNWreOIA/edit)**.**

- 1. **Consent to participate**
- **1. Chibvumirano kuva muchirongwa**

By clicking the boxes below, I confirm that:

Nekusarudza maBox ari pasi, ndinobvuma kuti:


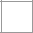


I have agreed to take part in the study

Ndabvuma kuva mutsvakurudzo


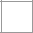


I have seen a copy of the information sheet (available by clicking the link above) that explains my role in this research. I understand its contents and agree to participate in this research.

Ndaona gwaro rinotaura nezvechirongwa (rinowanikwa nekubaya pamusoro apo) rinotsanangura zvandinotarisirwa kuva kwangu muchirongwa. Ndanzwisisa zvirimo uye nekubvuma kuva muchirongwa ichi.


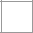


I can withdraw from the survey at any point in time

Ndinogona kuregera kupindura mibvunzo chero nguva ipi zvayo


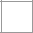


I will not have any financial benefits that result from the commercial development of this research

Handiwane mubairo wemari dzichabva pakubudirira kwetsvakurudzo iyi


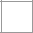


I consent to have the coded data made available for future research by putting it into a data repository

Ndinobvuma kuti mhinduro dzose dzakakwenenzverwa dzizoshandiswa kuzvirongwa zvichazouya dzichibva pakachengetedzwa

- 2. Thank you for your consent. If you provide text answers, do you agree for us to quote your statements (verbatim) in a report without identifying you?
- 2. Tinokutendai nekubvuma kuva muchirongwa. Kana muchinge manyora mhinduro, munobvuma here kuti tizonyora mhinduro yenyu semanyorero amunenge maita (Verbatim) mugwaro rinozobuda asi tisinganyore zita renyu?


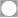
 Yes

Hongu


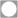
 No

Kwete

- 3. What is your age?
- 3. Makura zvakadii?
- 4. What is your gender?
- 4. Muri munhui?


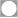
 Female

Mukadzi


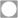
 Male

Murume


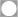
 Prefer not to answer

Ndinosarudza kusapindura


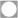
 Prefer to self describe:

Ndinosarudza kutaura zvandiri:

- 5. Which of the following best describes the role you work in?
- 5. Ndezvipi zviripasi apa zvinotaura zvamuri pabasa ramuri?


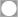
 Nurse providing care to patients

Mukoti anopa rubatsiro kuvarwere


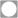
 Doctor providing care to patients

Chiremba anopa rubatsiro kuvarwere


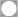
 Community healthcare worker

Mushandi wehutano munharaunda


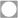
 Other healthcare provider

Mumwewo anopa rubatsiro nezveutano


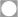
 Manager of healthcare facility or programme

Mutungamiri wepachipatara kana kuti wechirongwa


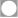
 Researcher

Mutsvakurudzi


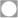
 Other (please specify)

Zvimwewo (tsanangura)


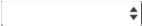

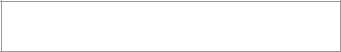

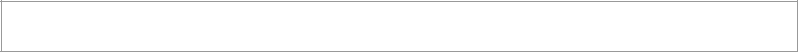


- 6. What type of organisation do you work in?
- 6. Sangano ramunoshandira rakaita sei?


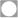
 Public sector healthcare facility

Chipatara chehurumende


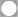
 **Private**, **for-profit** healthcare facility

Chipatara chakazvimiririra kuti chizowanawo mubairo wemari


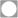
 **Charity**/**non-profit** healthcare facility

Chipatara chinorapa pachena


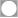
 Government agency

Bazi rehurumende


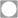
 Domestic non-governmental organisation

Sangano remunyika revakazvimiririra risiri pasi pehurumende


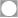
 International non-governmental organisation

Sangano rekunze revakazvimiririra risiri pasi pehurumende


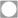
 Funding agency

Sangano renobatsira nezvemari


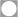
 University or academic body

University kana kuti gungano revezvefundo


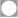
 Other (please specify)

Zvimwe wo (tsanangurai)

- 7. Which country are you providing information about?
- 7. Murikupa mhinduro nezvenyika ipi?
- 8. Please select whether you would like to answer questions on TB, HIV or both
- 8. Ndapota sarudzai kuti munoda kupindura nezveTB here kana kuti HIV kana zvese


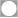
 TB

TB


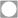
 HIV

HIV


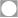
 Both

Zvese


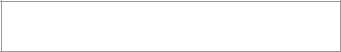

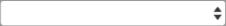


Please answer the 9 short questions on TB. Thank you!

Ndapota pindura mibvunzo mipfumbamwe nezveTB. Ndatenda!

**You can select 'prefer not to answer' for any questions you want to skip.**

**Muogona kusarudza ‘ndosarudza kusapindura’ pamibvunzo yamunoda kudarika.**

- 9. Has it been harder for **healthcare providers to come to work** at TB healthcare facilities since COVID-19?
- 9. Zvanga zvakambooma here kuti vashandi vezveutano vatadze kuuya kubasa kuzvipatara zveTB kubva pakatanga COVID-19?


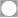
 No - same as before

Kwete – zvakangofanana nekare


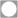
 Yes - it is slightly harder

Hongu – zvakati omei


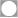
 Yes - it is much harder

Hongu - zvakaoma


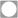
 Yes – it is very difficult or impossible

Hongu – zvakaomesesa kana kuti hazvitoite


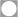
 Don’t know

Handzivi


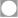
 Prefer not to answer

Ndosarudza kusapindura

- 10. Has it been harder for **TB patients to access TB services** since COVID-19?
- 10. Zvakambooma here kuti varwere veTB vawane rubatsiro kubva patanga COVID-19?


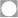
 No - same as before

Kwete – zvakangofanana nekare


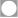
 Yes - it is slightly harder

Hongu – zvakati omei


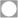
 Yes - it is much harder

Hongu - zvakaoma


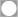
 Yes – it is very difficult or impossible

Hongu – zvakaomesesa kana kuti hazvitoite


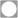
 Don’t know

Handzivi


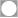
 Prefer not to answer

Ndosarudza kusapindura

- 11. What do you think are the main **concerns or barriers for TB patients** to access healthcare since COVID-19? (select all that apply)
- 11. Zvii zvamunofunga kuti zvingava zvichemo kana kuti zvinokonesa kuti varwere veTB vawane rubatsiro kubva patanga COVID-19? (sarudzai zvinoita zvese)


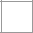


Physical distancing/lockdown rules

Kusava pedyo nepedyo kana kuti mitemo yezvekuvharwa kwezvinhu


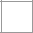


Disruptions to transport

Kukanganiswa kweafambiro emichovha


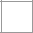


Reduced income/access to money to travel

Kuderedzwa kwemari yamunowana kana kushaya mari dzekufambisa


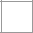


Fear of getting infected with COVID-19

Kutya kuzadzwa chirwere cheCOVID-19


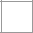


Closure of health facilities

Kuvharwa kwezvipatara


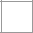


Healthcare provider shortages

Kushaikwa kwevanoita nezvehutano


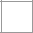


Longer waiting times

Nguva yakarebesa kuwana rubatsiro


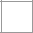


Unable to access a face mask

Kutadza kuwana face mask


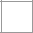


There are NO concerns or barriers for TB patients

Hapana zvichemo kana zvipingaidzo kuvarwere veTB


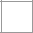


Prefer not to answer

Ndinosarudza kusapindura


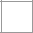


Other (please explain below)

Zimwewo (ndapota tsanangurai)


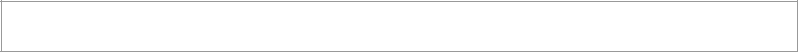


- 12. Since COVID-19, what **control measures have been implemented by the government** and how has this impacted TB health services? (examples: reduced transport, movement restrictions, etc)
- 12. Kubva patanga COVID-19, ndeipi mitemo yakaiswa nehurumende uye zvakabatsira kana kukanganisa sei mabasa eTB? (semuenzaniso: kushomeka kwemichovha, kusabvumidzwa kungofamba famba, etc)


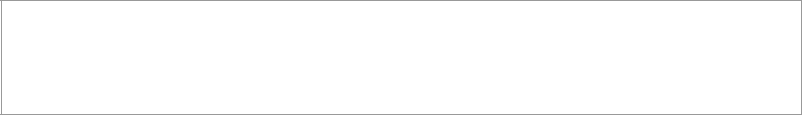


- 13. Since COVID-19, are you aware of any changes to the way **TB healthcare facilities are operating**? (select all that apply)
- 13. Kubva patanga COVID-19, munoziva here shanduko dzakaitika pamashandiro ekuzvipatara zveTB? (sarudzai zvese zvinoita)


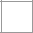


No - same as before

Kwete – zvakangofanana nekare


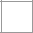


Yes – physical distancing protocols for patients

Hongu – mitemo yeku sava pedyo nepedyo kwevarwere


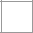


Yes – masks or other protective equipment for healthcare providers

Hongu – mamask kana kuti zvimwewo zvinodzivirira vashandi i vemuzvipatara


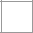


Prefer not to answer/ don't know

Ndinosarudza kusapindura/Handizivi


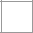


Yes - Other, please explain below

Hongu-Zvimwewo, ndapota tsanangurai pazasi


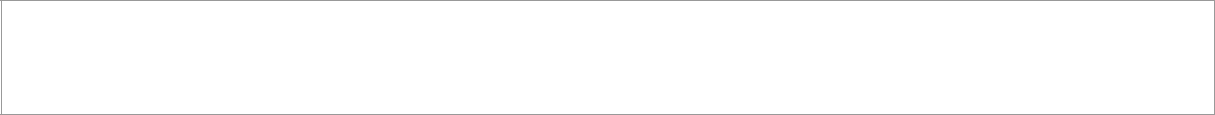


- 14. Have you experienced shortages of diagnostics or other challenges to provision of routine **diagnostic** **services** for TB since COVID-19?
- 14. Makamboshaya here zvinobatsira kuvheneka kana zvimhingamupinyi kumabasa ekuvhenekwa kweTB kubva patanga COVID-19?


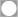
 No - same as before

Kwete – zvakangofanana nekare


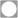
 Yes - it is slightly harder to provide diagnostic services

Hongu – zvakati womei kuita mabasa ekuvheneka zvirwere


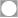
 Yes - it is much harder to provide diagnostic services

Hongu – zvakaoma kuita mabasa ekuvheneka zvirwere


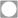
 Yes – it is very difficult or impossible to provide diagnostic services

Hongu – zvakaomesesa kana kusatoita mabasa ekuvheneka zvirwere


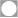
 Don’t know

Handizive


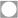
 Prefer not to answer

Ndinosarudza kusapindura

Please use this space to provide more details about what has caused the change

Ndapota shandisai nzvimbo iripasi kunyora zvizhinji zvakakonzera shanduko


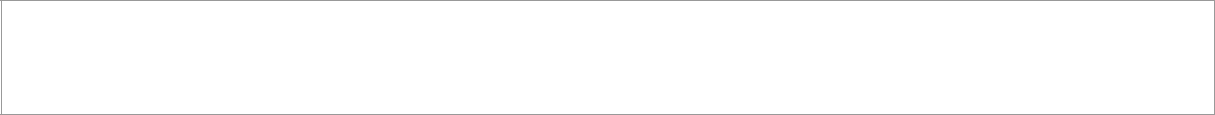


- 15. Have you experienced shortages of medicines or other challenges to provision of standard **treatment** for TB patients since COVID-19?
- 15. Makamboita dambuko rekushomeka kwemishonga here kana kuti matambudziko ekurapa kwakakwana kunotarisirwa kuvarwere veTB kubva patanga COVID-19?


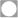
 No - same as before

Kwete- zvakangofanana nekare


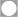
 Yes - it is slightly harder to provide TB treatment

Hongu – zvakati womei kurapa varwere veTB


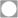
 Yes - it is much harder to provide TB treatment

Hongu – zvakati womei kurapa varwere veTB


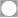
 Yes – it is very difficult or impossible to provide TB treatment

Hongu – zvakaomesesa kana kusatorapa varwere veTB


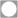
 Don’t know

Handiziva


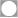
 Prefer not to answer

Ndinosarudza kusapindura

Please use this space to provide more details,

Ndapaota shandisai nzvimbo kunyora zvimwewo kusanganisira


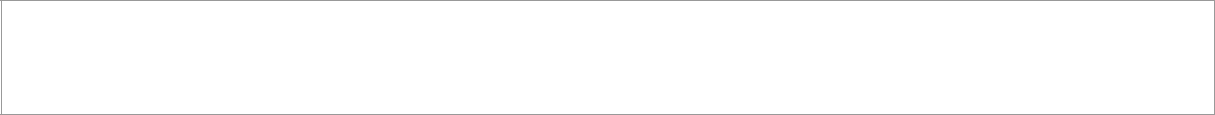


- 16. Has it been harder for TB patients to access **non-medical support** such as food supplementation or counselling since COVID-19?
- 16. Zvakange zvakambooma here kuti varwere veTB vatadze kuwana rumwewo rubatsiro rusiri kurapwa se kupihwa kwechikafu kana kupangwa mazano kubva patanga COVID-19?


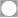
 No - same as before

Kwete- zvakangofanana nekare


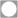
 Yes - it is slightly harder

Hongu zvakati womei


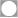
 Yes - it is much harder

Hogu- zvakaomesesa


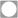
 Yes – it is very difficult or impossible

Hongu – zvakaomesesa kana kuti hazvitoite


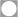
 Not available in my country, region, or facility

Hazviwanikwe munyika medu, nzvimbo, kana kuti chipatara


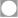
 Don’t know

Handizive


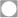
 Prefer not to answer

Ndinosarudza kusapindura

Please use this space to provide more details

Shandisai nzvimbo iripazasi kana munezvekuwedzera


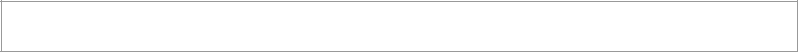


1. What do you think can be done (or has already been done) to **minimize or avoid disruptions from** **COVID-19** to TB services?

17. Chii chamunofunga kuti chingaitwe (kana kuti chakatoitwa) **kuderedza kana kuti kudzivirira zvinokanganisa mabasa eTB zvichikonzerwa neCOVID-19**?


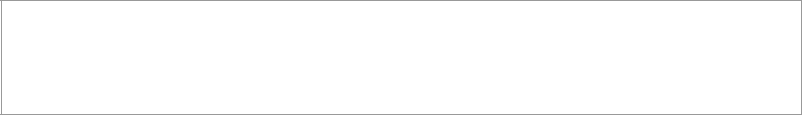


By clicking the **NEXT** button, you will end this survey. Please check your answers before continuing. Thank you for taking the time to answer this survey!

Pamunongobaya bhatani rakanzi **NEXT**, munobva mapedza mibvunzo iyoyi. Ndapota tarisai mhinduro dzenyu musati maenderera mberi. Tinokutendai nekutora nguva yenyu kupindura mibvunzo iyi!

Please answer the 9 short questions on HIV. Thank you!

Ndapota pindura mibvunzo mipfumbamwe nezveHIV. Ndatenda!

**You can select 'prefer not to answer' for any questions you want to skip.**

**Muogona kusarudza ‘ndosarudza kusapindura’ pamibvunzo yamunoda kudarika**

- 18. Has it been harder for **healthcare providers to come to work** at HIV healthcare facilities since COVID-19?
- 18. Zvanga zvakambooma here kuti vashandi vezveutano vatadze kuuya kubasa kuzvipatara zveHIV kubva pakatanga COVID-19?
- No - same as before
- Kwete – zvakangofanana nekare
-
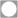
 Yes - it is slightly harder
- Hongu – zvakati omei
-
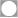
 Yes - it is much harder
- Hongu - zvakaoma
-
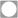
 Yes – it is very difficult or impossible
- Hongu – zvakaomesesa kana kuti hazvitoite
-
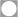
 Don’t know
- Handzivi
-
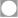
 Prefer not to answer
- Ndosarudza kusapindura
- 19. Has it been harder for **HIV patients to access HIV services** since COVID-19?
- . Zvakambooma here kuti varwere veHIV vawane rubatsiro kubva patanga COVID-19?


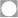
 No - same as before

- Kwete – zvakangofanana nekare


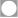
 Yes - it is slightly harder

- Hongu – zvakati omei


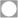
 Yes - it is much harder

- Hongu - zvakaoma

Yes – it is very difficult or impossible

- Hongu – zvakaomesesa kana kuti hazvitoite

Don’t know

Handizive

Prefer not to answer

Ndinosarudza kusapindura

- 20. What do you think are the main **concerns or barriers for HIV patients** to access healthcare since COVID -19?
- 20. Zvii zvamunofunga kuti zvingava zvichemo kana kuti zvinokonesa kuti varwere veHIV vawane rubatsiro kubva patanga COVID-19? (sarudzai zvinoita zvese)

Physical distancing/lockdown rules

Kusava pedyo nepedyo kana kuti mitemo yezvekuvharwa kwezvinhu

Disruptions to transport

Kukanganiswa kweafambiro emichovha

Reduced income/access to money to travel

Kuderedzwa kwemari yamunowana kana kushaya mari dzekufambisa

Fear of getting infected with COVID-19

Kutya kuzadzwa chirwere cheCOVID-19

Closure of health facilities

Kuvharwa kwezvipatara

Healthcare provider shortages

Kushaikwa kwevanoita nezvehutano

Longer waiting times

Nguva yakarebesa kuwana rubatsiro

Unable to access a face mask

Kutadza kuwana face mask

There are NO concerns or barriers for HIV patients

Hapana zvichemo kana zvipingaidzo kuvarwere veHIV

Prefer not to answer

Ndinosarudza kusapindura

Other (please explain below)

Zimwewo (ndapota tsanangurai)

- 21. Since COVID-19, what **control measures have been implemented by the government** and how has this impacted HIV health services? (examples: reduced transport, movement restrictions, etc)
- 21. Kubva patanga COVID-19, ndeipi mitemo yakaiswa nehurumende uye zvakabatsira kana kukanganisa sei mabasa eHIV? (semuenzaniso: kushomeka kwemichovha, kusabvumidzwa kungofamba famba, etc)

- 22. Since COVID-19, are you aware of any changes to the way **HIV healthcare facilities are** **operating**? (select all that apply)
- .22 Kubva patanga COVID-19, munoziva here shanduko dzakaitika pamashandiro ekuzvipatara zveHIV? (sarudzai zvese zvinoita)

No - same as before

Kwete – zvakangofanana nekare

Yes – physical distancing protocols for patients

Hongu – mitemo yeku sava pedyo nepedyo kwevarwere

Yes – masks or other protective equipment for healthcare providers

Hongu – mamask kana kuti zvimwewo zvinodzivirira vashandi i vemuzvipatara

Prefer not to answer/ don't know

Ndinosarudza kusapindura/Handizivi

Yes - Other, please explain below

Hongu-Zvimwewo, ndapota tsanangurai pazasi

- 23. Have you experienced shortages of diagnostics or other challenges to provision of routine **diagnostic** **services** for HIV since COVID-19?
- Makamboshaya here zvinobatsira kuvheneka kana zvimhingamupinyi kumabasa ekuvhenekwa kweHIV kubva patanga COVID-19?

No - same as before

Kwete – zvakangofanana nekare

Yes - it is slightly harder to provide diagnostic services

Hongu – zvakati womei kuita mabasa ekuvheneka zvirwere

Yes - it is much harder to provide diagnostic services

Hongu – zvakaoma kuita mabasa ekuvheneka zvirwere

Yes – it is very difficult or impossible to provide diagnostic services

Hongu – zvakaomesesa kana kusatoita mabasa ekuvheneka zvirwere

Don’t know

Handizive

Prefer not to answer

Ndinosarudza kusapindura

- Please use this space to provide more details about what has caused the change
- Ndapota shandisai nzvimbo iripasi kunyora zvizhinji zvakakonzera shanduko

- 24. Have you experienced shortages of medicines or other challenges to provision of standard **treatment** for HIV patients since COVID-19?
- 24. Makamboita dambuko rekushomeka kwemishonga here kana kuti matambudziko ekurapa kwakakwana kunotarisirwa kuvarwere veHIV kubva patanga COVID-19?

No - same as before

Kwete- zvakangofanana nekare

Yes - it is slightly harder to provide HIV treatment

Hongu – zvakati womei kurapa varwere veHIV

Yes - it is much harder to provide HIV treatment

Hongu – zvakati womei kurapa varwere veHIV

Yes – it is very difficult or impossible to provide TB treatment

Hongu – zvakaomesesa kana kusatorapa varwere veHIV

Don’t know

Handiziva

Prefer not to answer

Ndinosarudza kusapindura

Please use this space to provide more details

Ndapota shandisai nzvimbo kunyora zvimwewo

- 25. Has it been harder for HIV patients to access **non-medical support** such as food supplementation or counselling since COVID-19?
- . Zvakange zvakambooma here kuti varwere veHIV vatadze kuwana rumwewo rubatsiro rusiri kurapwa se kupihwa kwechikafu kana kupangwa mazano kubva patanga COVID-19?

No - same as before

Kwete- zvakangofanana nekare

Yes - it is slightly harder

Hongu zvakati womei

Yes - it is much harder

Hogu- zvakaomesesa

Yes – it is very difficult or impossible

Hongu – zvakaomesesa kana kuti hazvitoite

Not available in my country, region, or facility

Hazviwanikwe munyika medu, nzvimbo, kana kuti chipatara

Don’t know

Handizive

Prefer not to answer

Ndinosarudza kusapindura

Please use this space to provide more details

Shandisai nzvimbo iripazasi kana munezvekuwedzera

1. What do you think can be done (or has already been done) to **minimize or avoid disruptions from** **COVID-19** to HIV services?

26. Chii chamunofunga kuti chingaitwe (kana kuti chakatoitwa) **kuderedza kana kuti kudzivirira zvinokanganisa mabasa eHIV zvichikonzerwa neCOVID-19**?

By clicking the **NEXT** button, you will end this survey. Please check your answers before continuing. Thank you for taking the time to answer this survey!

Pamunongobaya bhatani rakanzi **NEXT**, munobva mapedza mibvunzo iyoyi. Ndapota tarisai mhinduro dzenyu musati maenderera mberi. Tinokutendai nekutora nguva yenyu kupindura mibvunzo iyi!
