# Supplementary material for: Mitigating the impact of COVID-19 on tuberculosis and HIV services: A cross-sectional survey of 669 health professionals in 64 low and middle-income countries
Source: PLoS One. 2021 Feb 2;16(2):e0244936. doi: 10.1371/journal.pone.0244936 (PMC7853462; doi:10.1371/journal.pone.0244936)
Supplement: S1 File — (ZIP) [file pone.0244936.s001.zip › Simplified_Chinese_JH .docx]

Identifying and mitigating impact of COVID-19 on TB and HIV programmes

识别及减低2019冠状病毒对于结核病和艾滋病项目的影响

Information 资讯

**We are conducting a short survey to understand ways in which TB and HIV services have been impacted by COVID-19 in low and middle income countries**

**我们正在进行简短的问卷调查，以了解2019冠状病毒（COVID-19）如何影响低收入和中收入国家的结核病及艾滋病服务。**

**The results will help to identify ways to protect and improve TB and HIV services
研究的结果将会有助于寻找出对结核病和艾滋病服务的保护及改善方法。**

**This survey is for people who are involved in managing or delivering TB or HIV services (doctors, nurses, policymakers, health facility managers, community groups and researchers). The survey is not intended for patients.**

**这份问卷的研究对象是正在管理或提供有关结核病或艾滋病服务的人士（医生，护士，政策制定者，医疗机构管理人员，社区组织，以及研究员）。这份问卷并不是为病人而设。**

**You do not have to provide your name or any other details that will allow answers to be traced back to you. All information will be kept completely anonymous.**

**您不需要提供您的姓名或其他任何会曝露您身份的作答细节。所有资料将会完全匿名储存。**

**Depending on your area of work, you can answer questions about TB (approximately 15 minutes) or HIV (approximately 15 minutes) or both.**

**您可以根据您工作的领域，回答有关结核病的部分（约15分钟）或艾滋病的部分（约15分钟），或两者都回答。**

**Once you start the survey you will need to complete it. You cannot save and come back, so please start the survey when you have enough time (15-30 minutes).**

**一旦开始回答问卷, 请一气呵成。您将不能储存部分答案然后继续回答，因此敬请您预留充足的时间来完成问卷（约15至30分钟）。**

**Please do not answer this survey more than once.**

**请勿回答这份问卷多于一次。**

**Detailed information about the study and your participation is available to download by clicking** [**here.**](https://docs.google.com/document/d/1L1MrsHnQUj1V72LJV2cYHe7oJlKA_OIAS1gkNWreOIA/edit)

**您可以在这里下载有关这项研究以及您参与的详情。**

- 1. **Consent to participate**同意参与

By clicking the boxes below, I confirm that:
透过按以下方格，我确认：

I have agreed to take part in the study

我同意参与这项研究

I have seen a copy of the information sheet (available by clicking the link above) that explains my role in this research. I understand its contents and agree to participate in this research.

我已经详读“参与者须知”（可从上面连接下载），包括有关我在这项研究的角色。我明白当中内容，并同意参与研究。

I can withdraw from the survey at any point in time

我可以随时停止参与这项研究调查。

I will not have any financial benefits that result from the commercial development of this research

我不会从这项研究的商业研发而获取任何经济利益。

I consent to have the coded data made available for future research by putting it into a data repository

我同意将编码后的数据储存在数据库，以便将来研究使用。

- 2. Thank you for your consent. If you provide text answers, do you agree for us to quote your statements (verbatim) in a report without identifying you?
  感谢您同意参与研究。如果您提供文字答案，您是否同意我们在不会披露您身份的情况下于报告中引用您的答案？

Yes

是

No

否

- 3. What is your age?
  您的年龄是？
- 4. What is your gender?
  您的性别是？

Female
女性

Male

男性

Prefer not to answer

不作回答

Prefer to self describe:
自我形容：

- 5. Which of the following best describes the role you work in?
  以下哪项最能够形容您的工作？

Nurse providing care to patients

照料病人的护士

Doctor providing care to patients

照料病人的医生

Community healthcare worker

社区医疗保健人员

Other healthcare provider

其他医疗服务人员

Manager of healthcare facility or programme

医疗机构或项目的管理人员

Researcher

研究员

Other (please specify)

其他（请註明）

- 6. What type of organisation do you work in?
  您在哪一种机构工作？

Public sector healthcare facility

公营医疗机构

**Private**, **for-profit** healthcare facility

**私营，牟利**医疗机构

**Charity**/**non-profit** healthcare facility

**慈善/非牟利**医疗机构

Government agency

政府单位

Domestic non-governmental organisation

国内非政府机构

International non-governmental organisation

国际非政府机构

Funding agency

提供资助的机构/基金会

University or academic body

大学或学术单位

Other (please specify)

其他（请註明）

- 7. Which country are you providing information about?
  您提供的资料是有关哪一个国家？
- 8. Please select whether you would like to answer questions on TB, HIV or both
  请选择您将回答的是有关结核病，艾滋病，或着是两者的题目

TB

结核病

HIV

艾滋病

Both

两者

Please answer the 9 short questions on TB. Thank you!

请您回答有关结核病的9条简短题目。谢谢！

**You can select 'prefer not to answer' for any questions you want to skip.**

**您可以在不想回答的题目选择“不作回答“。**

- 9. Has it been harder for **healthcare providers to come to work** at TB healthcare facilities since COVID-19?
  自从2019冠状病毒的疫情以来，**医疗服务人员到结核病医疗单位上班**是否变得更加困难？

No - same as before

否-跟之前一样

Yes - it is slightly harder

是-变得困难一点

Yes - it is much harder

是-变得困难很多

Yes – it is very difficult or impossible

是-变得非常困难或没有可能

Don’t know

不知道

Prefer not to answer

不作回答

- 10. Has it been harder for **TB patients to access TB services** since COVID-19?
  自从2019冠状病毒的疫情以来，**结核病病人在获取结核病医疗服务**上是否变得更加困难？

No - same as before
否-跟之前一样

Yes - it is slightly harder

是-变得困难一点

Yes - it is much harder

是-变得困难很多

Yes – it is very difficult or impossible

是-变得非常困难或没有可能

Don’t know

不知道

Prefer not to answer

不作回答

- 11. What do you think are the main **concerns or barriers for TB patients** to access healthcare since COVID-19? (select all that apply)
  自从2019冠状病毒的疫情以来，您认为**结核病病人**在获取结核病的医疗照护上的**主要困难或障碍**是什么？(可复选)

Physical distancing/lockdown rules

肢体距离/封锁规定

Disruptions to transport

交通影响

Reduced income/access to money to travel

收入减少/用于交通的资金减少

Fear of getting infected with COVID-19

担心感染2019冠状病毒

Closure of health facilities

医疗设施关闭

Healthcare provider shortages

医疗人员不足

Longer waiting times

等候时间加长

Unable to access a face mask

不能获取口罩

There are NO concerns or barriers for TB patients

没有困难或障碍

Prefer not to answer

不作回答

Other (please explain below)

其他（请于以下解释）

- 12. Since COVID-19, what **control measures have been implemented by the government** and how has this impacted TB health services? (examples: reduced transport, movement restrictions, etc)

自从2019冠状病毒的疫情以来，**政府实施了哪些控制措施**，而这些措施对结核病的医疗照护又有何影响？（例如：减少交通，限制人群流动，等）

- 13. Since COVID-19, are you aware of any changes to the way **TB healthcare facilities are operating**? (select all that apply)

自从2019冠状病毒的疫情以来，您是否知道**结核病医疗机构在营运方面**上有否任何改变？（可复选）

No - same as before

否-跟之前一样

Yes – physical distancing protocols for patients

是-病人需按照肢体距离规则

Yes – masks or other protective equipment for healthcare providers

是-医疗人员佩戴口罩或其他保护装备

Prefer not to answer/ don't know

不作回答/不知道

Yes - Other, please explain below

是-其他，请于以下解释

- 14. Have you experienced shortages of diagnostics or other challenges to provision of routine **diagnostic** **services** for TB since COVID-19?
  自从2019冠状病毒的疫情以来，您是否经历过缺乏诊断仪器或因遇到其他困难而无法提供常规的结核病**诊断服务**？

No - same as before

否-跟之前一样

Yes - it is slightly harder to provide diagnostic services

是-提供诊断服务变得困难一点

Yes - it is much harder to provide diagnostic services

是-提供诊断服务变得困难很多

Yes – it is very difficult or impossible to provide diagnostic services

是-提供诊断服务变得非常困难或没有可能

Don’t know

不知道

Prefer not to answer

不作回答

Please use this space to provide more details about what has caused the change

请在空格填写有关产生这种改变的原因

- 15. Have you experienced shortages of medicines or other challenges to provision of standard **treatment** for TB patients since COVID-19?
  自从2019冠状病毒的疫情以来，您是否经历过缺乏药物或因遇到其他困难而无法提供结核病病人的一般**治疗**？

No - same as before

否-跟之前一样

Yes - it is slightly harder to provide TB treatment

是-提供结核病治疗服务变得困难一点

Yes - it is much harder to provide TB treatment

是-提供结核病治疗服务变得困难很多

Yes – it is very difficult or impossible to provide TB treatment

是-提供结核病治疗服务变得非常困难或没有可能

Don’t know

不知道

Prefer not to answer

不作回答

Please use this space to provide more details

请在空格填写有关详情

- 16. Has it been harder for TB patients to access **non-medical support** such as food supplementation or counselling since COVID-19?
  自从2019冠状病毒的疫情以来，结核病病人在获取**非医疗援助**（例如补充食物或辅导服务）上是否遇到更大的困难？

No - same as before

否-跟之前一样

Yes - it is slightly harder

是-变得困难一点

Yes - it is much harder

是-变得困难很多

Yes – it is very difficult or impossible

是-变得非常困难或没有可能

Not available in my country, region, or facility

在我的国家，地区，或设施没有提供

Don’t know

不知道

Prefer not to answer

不作回答

Please use this space to provide more details

请在空格填写有关详情

1. What do you think can be done (or has already been done) to **minimize or avoid disruptions from** **COVID-19** to TB services?

您认为有什么能够（或已经）**减低或避免2019冠状病毒对于结核病服务的影响**？

By clicking the **NEXT** button, you will end this survey. Please check your answers before continuing. Thank you for taking the time to answer this survey!

按下“继续”后，您将会结束这份问卷。请先核对您的答案。感谢您抽出宝贵时间参与这项研究！

Please answer the 9 short questions on HIV. Thank you!

请您回应有关艾滋病的9条简短题目。谢谢！

**You can select 'prefer not to answer' for any questions you want to skip.**

**您可以在不想回答的题目选择“不作回答“**

- 18. Has it been harder for **healthcare providers to come to work** at HIV healthcare facilities since COVID-19?
- 自从2019冠状病毒的疫情以来，**医疗服务人员到艾滋病医疗单位上班**是否变得更加困难？

No - same as before

否-跟之前一样

Yes - it is slightly harder

是-变得困难一点

Yes - it is much harder

是-变得困难很多

Yes – it is very difficult or impossible

是-变得非常困难或没有可能

Don’t know

不知道

Prefer not to answer

不作回答

- 19. Has it been harder for **HIV patients to access HIV services** since COVID-19?
  自从2019冠状病毒的疫情以来，**艾滋病病人在获取艾滋病医疗服务**上是否变得更加困难？

No - same as before

否-跟之前一样

Yes - it is slightly harder

是-变得困难一点

Yes - it is much harder

是-变得困难很多

Yes – it is very difficult or impossible

是-变得非常困难或没有可能

Don’t know

不知道

Prefer not to answer

不作回答

- 20. What do you think are the main **concerns or barriers for HIV patients** to access healthcare since COVID -19?
  自从2019冠状病毒的疫情以来，您认为**艾滋病病人**在获取艾滋病的医疗照护上的**主要困难或障碍**是什么？(可复选)

Physical distancing/lockdown rules

肢体距离/封锁规定

Disruptions to transport

交通影响

Reduced income/access to money to travel

收入减少/用于交通的资金减少

Fear of getting infected with COVID-19

担心感染2019冠状病毒

Closure of health facilities

医疗设施关闭

Healthcare provider shortages

医疗人员不足

Longer waiting times

等候时间加长

Unable to access a face mask

不能获取口罩

There are NO concerns or barriers for HIV patients at this time

没有困难或障碍

Prefer not to answer

不作回答

Other (please explain below)

其他（请于以下解释）

- 21. Since COVID-19, what **control measures have been implemented by the government** and how has this impacted HIV health services? (examples: reduced transport, movement restrictions, etc)

自从2019冠状病毒的疫情以来，**政府实施了哪些控制措施**，而这些措施对艾滋病的医疗照护又有何影响？

- 22. Since COVID-19, are you aware of any changes to the way **HIV healthcare facilities are** **operating**? (select all that apply)
  自从2019冠状病毒的疫情以来，您是否知道**艾滋病医疗设施在营运方式**上有否任何改变？（可复选）

No - same as before

否-跟之前一样

Yes – physical distancing protocols for patients

是-病人需按照肢体距离规则

Yes – masks or other protective equipment for healthcare providers

是-医疗人员佩戴口罩或其他保护装备

Prefer not to answer/ don't know

不作回答/不知道

Yes- Other (please explain below)

是-其他，请于以下解释

- 23. Have you experienced shortages of diagnostics or other challenges to provision of routine **diagnostic** **services** for HIV since COVID-19?
  自从2019冠状病毒的疫情以来，您是否经历过缺乏诊断仪器或因遇到其他困难而无法提供常规的艾滋病**诊断服务**？

No - same as before

否-跟之前一样

Yes - it is slightly harder to provide diagnostic services

是-提供诊断服务变得困难一点

Yes - it is much harder to provide diagnostic services

是-提供诊断服务变得困难很多

Yes – it is very difficult or impossible to provide diagnostic services

是-提供诊断服务变得非常困难或没有可能

Don’t know

不知道

Prefer not to answer

不作回答

Please use this space to provide more details:

请在空格填写有关详情

- 24. Have you experienced shortages of medicines or other challenges to provision of standard **treatment** for HIV patients since COVID-19?
  自从2019冠状病毒的疫情以来，您是否经历过缺乏药物或因遇到其他困难而无法提供艾滋病病人的一般**治疗?**

No - same as before

否-跟之前一样

Yes - it is slightly harder to provide HIV treatment

是-提供艾滋病病毒治疗服务变得困难一点

Yes - it is much harder to provide HIV treatment

是-提供艾滋病病毒治疗服务变得困难很多

Yes – it is very difficult or impossible to provide HIV treatment

是-提供艾滋病病毒治疗服务变得非常困难或没有可能

Don’t know

不知道

Prefer not to answer

不作回答

Please use this space to provide more details:

请在空格填写有关详情：

- 25. Has it been harder for HIV patients to access **non-medical support** such as food supplementation or counselling since COVID-19?
  自从2019冠状病毒的疫情以来，艾滋病病人在获取**非医疗援助**（例如补充食物或辅导服务）上是否遇到更大困难？

No - same as before

否-跟之前一样

Yes - it is slightly harder

是-变得困难一点

Yes - it is much harder

是-变得困难很多

Yes – it is very difficult or impossible

是-变得非常困难或没有可能

Not available in my country, region, or facility

在我的国家，地区，或设施没有提供

Don’t know

不知道

Prefer not to answer

不作回答

Please use this space to provide more details

请在空格填写有关详情

1. What do you think can be done (or has already been done) to **minimize or avoid disruptions from** **COVID-19** to HIV services?
   您认为有什么能够（或已经）**减低或避免2019冠状病毒对于艾滋病服务的影响**？

By clicking the **NEXT** button, you will end this survey. Please check your answers before continuing. Thank you for taking the time to answer this survey!
按下“继续”后，您将会结束这份问卷。请先核对您的答案。感谢您抽出宝贵时间参与这项研究！

**There will first be 9 short questions about TB, followed by 9 short questions about HIV.
接下来将会先有9条有关结核病的简短题目，之后将有9条有关艾滋病的简短题目。**

**You can select 'prefer not to answer' for any questions you want to skip.
您可以在不想回答的题目选择“不作回答“。**

**Thank you for your time!**

**感谢您的宝贵时间！**

- 27. Has it been harder for **healthcare providers to come to work** at TB healthcare facilities since COVID-19?

No - same as before

Yes - it is slightly harder

Yes - it is much harder

Yes – it is very difficult or impossible

Don’t know

Prefer not to answer

- 28. Has it been harder for **TB patients to access TB services** since COVID-19?

No - same as before

Yes - it is slightly harder

Yes - it is much harder

Yes – it is very difficult or impossible

Don’t know

Prefer not to answer

- 29. What do you think are the main **concerns or barriers** for TB patients to access healthcare since COVID-19? (select all that apply)

Physical distancing/lockdown rules

Disruptions to transport

Reduced income/access to money to travel

Fear of getting infected with COVID-19

Closure of health facilities

Healthcare provider shortages

Longer waiting times

Unable to access a face mask

There are NO concerns or barriers for TB patients

Prefer not to answer

Other (please specify)

- 30. Since COVID-19, what **control measures have been implemented by the government** and how has this impacted TB health services? (examples: reduced transport, movement restrictions, etc)

- 31. Since COVID-19, are you aware of any changes to the way **TB healthcare facilities are** **operating**? (select all that apply)

No - same as before

Yes – physical distancing protocols for patients

Yes – masks or other protective equipment for healthcare providers

Prefer not to answer/don't know

Yes- Other. Please explain below

- 32. Have you experienced shortages of diagnostics or other challenges to provision of routine **diagnostic** **services** for TB since COVID-19?

No - same as before

Yes - it is slightly harder to provide diagnostic services

Yes - it is much harder to provide diagnostic services

Yes – it is very difficult or impossible to provide diagnostic services

Don’t know

Prefer not to answer

Please use this space to provide more details about what has caused the change:

- 33. Have you experienced shortages of medicines or other challenges to provision of standard **treatment** for TB patients since COVID-19?

No - same as before

Yes - it is slightly harder to provide TB treatment

Yes - it is much harder to provide TB treatment

Yes – it is very difficult or impossible to provide TB treatment

Don’t know

Prefer not to answer

Please use this space to provide more details

34. Has it been harder for TB patients to access **non-medical support** such as food supplementation or counselling since COVID-19?

No - same as before

Yes - it is slightly harder

Yes - it is much harder

Yes – it is very difficult or impossible

Not available in my country, region, or facility

Don’t know

Prefer not to answer

Please use this space to provide more details

1. What do you think can be done (or has already been done) to **minimize or avoid disruptions from** **COVID-19** to TB services?

Please answer the following 9 questions on HIV

**You can select 'prefer not to answer' for any questions you want to skip.**

- 36. Has it been harder for **healthcare providers to come to work** at HIV healthcare facilities since COVID-19?

No - same as before

Yes - it is slightly harder

Yes - it is much harder

Yes – it is very difficult or impossible

Don’t know

Prefer not to answer

37. Has it been harder for **HIV patients to access HIV services** since COVID-19?

No - same as before

Yes - it is slightly harder

Yes - it is much harder

Yes – it is very difficult or impossible

Don’t know

Prefer not to answer

- 38. What do you think are the main **concerns or barriers for HIV patients** to access healthcare since COVID -19?

Physical distancing/lockdown rules

Disruptions to transport

Reduced income/access to money to travel

Fear of getting infected with COVID-19

Closure of health facilities

Healthcare provider shortages

Longer waiting times

Unable to access a face mask

There are NO concerns or barriers for TB patients

Prefer not to answer/don't know

Other (please specify)

- 39. Since COVID-19, what **control measures have been implemented by the government** and how has this impacted HIV health services? (examples: reduced transport, movement restrictions, etc)

- 40. Since COVID-19, are you aware of any changes to the way **HIV healthcare facilities** are operating? (select all that apply)

No - same as before

Yes – physical distancing protocols for patients

Yes – masks or other protective equipment for healthcare providers

Prefer not to answer/don't know

Yes- Other

- 41. Have you experienced shortages of diagnostics or other challenges to provision of routine **diagnostic** **services** for HIV since COVID-19?

No - same as before

Yes - it is slightly harder to provide diagnostic services

Yes - it is much harder to provide diagnostic services

Yes – it is very difficult or impossible to provide diagnostic services

Don’t know

Prefer not to answer

Please use this space to provide more details about what has caused the change:

- 42. Have you experienced shortages of medicines or other challenges to provision of standard **treatment** for HIV patients since COVID-19?

No - same as before

Yes - it is slightly harder to provide HIV treatment

Yes - it is much harder to provide HIV treatment

Yes – it is very difficult or impossible to provide HIV treatment

Don’t know

Prefer not to answer

Please use this space to provide more details:

- 43. Has it been harder for HIV patients to access **non-medical support** such as food supplementation or counselling since COVID-19?

No - same as before

Yes - it is slightly harder

Yes - it is much harder

Yes – it is very difficult or impossible

Not available in my country, region, or facility

Don’t know

Prefer not to answer

Please use this space to provide more details

1. What do you think can be done (or has already been done) to **minimize or avoid disruptions from** **COVID-19** to HIV services?

By clicking the **DONE** button, you will end this survey. Please check your answers before continuing. Thank you for taking the time to answer this survey!
