# Supplementary material for: Mitigating the impact of COVID-19 on tuberculosis and HIV services: A cross-sectional survey of 669 health professionals in 64 low and middle-income countries
Source: PLoS One. 2021 Feb 2;16(2):e0244936. doi: 10.1371/journal.pone.0244936 (PMC7853462; doi:10.1371/journal.pone.0244936)
Supplement: S1 File — (ZIP) [file pone.0244936.s001.zip › Spanish.docx]

Identificación y mitigación del impacto de la COVID-19 en programas de TB y VIH.

Información

**Estamos realizando una breve encuesta, en países de ingresos medios y bajos, para entender la forma en que la COVID-19 ha impactado en los servicios de TB y VIH.**

**Los resultados nos ayudaran a identificar estrategias para proteger y mejorar los servicios de TB y VIH.**

**Esta encuesta va dirigida a aquellas personas que proporcionan y gestionan servicios relacionados con TB y VIH (médicas/os, enfermeras/os, responsables políticos, gestores de centros sanitarios , organizaciones comunitarias e investigadores/as). La encuesta no va dirigida a pacientes.**

**No es necesario que nos proporcione su nombre o cualquier otro dato que le pueda identificar. Toda la información se mantendrá en el más estricto anonimato.**

**Dependiendo de su área de trabajo, puede contestar a las preguntas relacionadas con la TB (le llevará aproximadamente 15 minutos) o el VIH (aproximadamente 15 minutos) o ambos.**

**Una vez que comience a cumplimentar la encuesta deberá completarla. No se podrá guardar y retomar, así que por favor empiece cuando tenga tiempo suficiente (15 - 30 minutos).**

**Por favor, no responda a esta encuesta más de una vez.**

**La información detallada sobre el estudio y sobre su participación se encuentra disponible para descargar en este** [**enlace.**](https://docs.google.com/document/d/1oIzlsv2gG1t6JP_snwJZnfmZ777n9TO7U2G_Xw3Te4c/edit?usp=sharing)

1. **Consentimiento para participar**

Señalando las siguientes casillas, confirmo que:

Estoy de acuerdo con participar en la encuesta.

He leído una copia de la hoja de información (disponible en el enlace anterior) en la que se describe mi papel en esta investigación. Entiendo su contenido y estoy de acuerdo con participar.

Me puedo salir de la encuesta en cualquier momento

No obtendré ningún beneficio económico como resultado del desarrollo comercial de esta investigación.

Doy mi consentimiento para que los datos codificados estén disponibles para futuros estudios y que para ello se almacenen en un repositorio de datos..

2. Gracias por su consentimiento. Si usted contesta con respuestas de texto, ¿nos permite citar sus respuestas (literales) en un informe sin que le identifiquemos?

Sí

No

3. ¿Qué edad tiene?

4. ¿Cuál es su género?

Femenino

Masculino

Prefiero no contestar

Prefiero autodefinirme

5. ¿Cuál de las siguientes opciones describe mejor su rol en el trabajo que desempeña?

Enfermera/o atendiendo a pacientes

Médica/o atendiendo a pacientes

Trabajador/a sanitario comunitario

Otro tipo de personal sanitario

Gestor/a de centro o programasanitario

Investigador/a

Otro (por favor especifique)

6. ¿En qué tipo de organización trabaja?

Servicio de salud público

**Servicio de salud privadoy lucrativo.**

Centro de salud sin ánimo de lucro

Agencia gubernamental

Organización local no gubernamental

Organización internacional no gubernamental

Agencia de financiación

Organismo universitario o académico

Otro (por favor especifique)

7. ¿Sobre qué país está usted proporcionando información?

8. Por favor seleccione si quiere contestar a las preguntas sobre TB, VIH o ambos

TB

VIH

Ambos

Por favor responda a las 9 preguntas sobre TB.

**Puede seleccionar “prefiero no contestar” para cualquier pregunta que quiera saltarse.**

9. ¿Desde la COVID-19, ha sido más difícil para los trabajadores sanitarios acudir a trabajar a los centros de TB?

No - igual que antes

Sí - un poco más difícil

Sí - bastante más difícil

Sí - es muy difícil o imposible

No sé

Prefiero no contestar

10. ¿Desde la COVID-19,ha sido más difícil para los pacientes de TB, acceder a los servicios de TB?

No – igual que antes

Sí – un poco más difícil

Sí – bastante más difícil

Sí – es muy difícil o imposible

No sé

Prefiero no contestar

11. ¿Desde la COVID-19, cuáles cree que son las principales preocupaciones o dificultades que tienen los pacientes con TB para acceder a los servicios sanitarios? (puede seleccionar más de una opción)

Distanciamiento físico/ Normas de confinamiento

Alteraciones en el transporte

Reducción de ingresos/Falta de recursos económicos para el transporte

Miedo al contagio por COVID-19

Cierre de los centros sanitarios

Escasez de recursos en los servicios sanitarios

Tiempos de espera más largos

Imposibilidad de acceso a mascarillas

No existen preocupaciones o dificultades entre los pacientes con TB

Prefiero no contestar

Otro (por favor explíquelo a continuación)

12. ¿Desde la COVID-19, qué medidas de control ha implementado el gobierno y de qué manera han impactado estas medidas en los servicios sanitarios para la TB? (ejemplos: reducción del transporte, restricciones de movilidad, etc)

13. ¿Desde la COVID-19, sabe si se ha producido algún cambio en la forma de funcionar de los centros sanitarios para la TB?(puede elegir más de una opción)

No – igual que antes

Sí – protocolos de distanciamiento físico para pacientes

Sí– mascarillas y otras medidas de protección para el personal sanitario.

Prefiero no contestar/ no sé

Sí - Otros, por favor explíquelo a continuación

14. ¿Desde la COVID-19, ha experimentado alguna falta de recursos diagnósticos u otros problemas con la provisión de servicios diagnósticos rutinarios para la TB?

No – igual que antes

Sí - es algo más difícil proporcionar servicios diagnósticos

Sí - es bastante más difícil proporcionar servicios diagnósticos

Sí - es muy difícil o imposible proporcionar servicios diagnósticos

No sé

Prefiero no contestar

Por favor utilice este espacio para dar más detalles sobre las causas de esoscambios.

15. ¿Desde el COVID-19, ha experimentado escasez de medicinas o algún otro problema en la provisión del tratamiento estándar para los pacientes con TB?

No – igual que antes

Sí – es algo más difícil proporcionar tratamiento para pacientes con TB

Sí – es bastante más difícil proporcionar tratamiento para pacientes con TB

Sí - es muy difícil o imposible proporcionar tratamiento para pacientes con TB

No sé

Prefiero no contestar

Por favor utilice este espacio para dar más detalles,incluyendo posibles dificultades en el suministro de antirretrovirales para pacientes con TB)

l

16. ¿Desde la COVID-19, ha sido más difícil para los pacientes con TB el acceso al apoyo no-médico, tales como suplementos alimenticios o asesoramiento médico?

No – igual que antes

Sí - algo más difícil

Sí -bastante más difícil

Sí – es muy difícil o imposible.

No está disponible en mi país, región o servicios sanitarios

No sé

Prefiero no contestar

Por favor utilice este espacio para dar más detalles.

1. ¿Qué piensa que se pueda hacer (o que ya se haya hecho) para minimizar o evitar los problemas de funcionamiento debidos a la COVID-19 en los servicios de TB?

Haciendo clic en el siguiente botón, habrá terminado esta encuesta. Por favor compruebe sus respuestas antes de continuar. Gracias por su colaboración.

Por favor conteste a las 9 preguntas breves sobre VIH. ¡Muchas gracias!

**Puede seleccionar “prefiero no contestar” para cualquier pregunta que quiera saltarse.**

18. ¿Desde la COVID-19, ha sido más difícil para los trabajadores sanitarios acudir a sus puestos de trabajo en los servicios de VIH?

No – igual que antes

Sí - algo más difícil

Sí - bastante más difícil

Sí -es muy difícil o imposible

No sé

Prefiero no contestar

19. ¿Desde la COVID-19, ha sido más difícil para los pacientes de VIH el acceso a los servicios de VIH?

No – igual que antes

Sí – es algo más difícil

Sí - bastante más difícil

Sí - es muy difícil o imposible

No sé

Prefiero no contestar

20. ¿Desde la COVID-19, qué dificultades o problemas deben sortear los pacientes con VIH para acceder a los servicios sanitarios?

Distanciamiento físico / normas de confinamiento

Alteraciones del transporte/restricción de la movilidad

Reducción de ingresos/Falta de recursos económicos para el transporte

Miedo al contagio con COVID-19

El cierre de los servicios sanitarios

Escasez de recursos sanitarios

Tiempos de espera más largos

Imposibilidad de acceso a mascarillas

No existen preocupaciones o barreras entre los pacientes con VIH.

Prefiero no contestar

Otro (por favor explíquelo a continuación)

21. ¿Desde la COVID-19, qué medidas de control ha implementado el gobierno y como han impactado en los servicios sanitarios de VIH? (por ejemplo: reducción del transporte, restricciones de movilidad, etc).

22. ¿Desde la COVID-19, se ha producido algún cambio en la forma de funcionar de los servicios sanitarios de VIH? (seleccione todo lo relacionado).

No – igual que antes

Sí – protocolos de distanciamiento físico para los pacientes

Sí – mascarillas y otras medidas de protección para los trabajadores sanitarios

Prefiero no contestar/no sé

Sí -otros (explíquelo a continuación)

23. ¿ Desde la COVID-19, ha experimentado alguna falta de recursos diagnósticos u otros problemas con la provisión de servicios diagnósticos rutinarios para el VIH?

No – igual que antes

Sí - es algo más difícil proporcionar servicios de diagnóstico

Sí -es bastante más difícil proporcionar servicios de diagnóstico

Sí - es muy difícil o imposible proporcionar servicios de diagnóstico

No sé

Prefiero no contestar

Por favor utilice este espacio para dar más detalles:

24. ¿Desde la COVID-19, ha tenido escasez de medicinas o algún otro problema en la provisión del tratamiento estándar para los pacientes con VIH?

No – Igual que antes

Sí – es algo más difícil proporcionar tratamiento para los pacientes con VIH

Sí – es bastante más difícil proporcionar tratamiento para los pacientes con VIH

Sí – es muy difícil o imposible proporcionar tratamiento para los pacientes con VIH

No sé

Prefiero no contestar

Por favor utilice este espacio para dar más detalles:

25. ¿Desde la COVID-19, ha sido más difícil para los pacientes con VIH el acceso al apoyo no-médico, tales como suplementos alimenticios o asesoramiento médico?

No – igual que antes

Sí – algo más difícil

Sí - bastante más difícil

Sí - es muy difícil o imposible

No está disponible en mi país, región o servicio sanitario.

No sé

Prefiero no contestar

Por favor utilice este espacio para dar más detalles

1. ¿Qué piensa que se pueda hacer (o que ya se haya hecho) para minimizar o evitar los problemas de funcionamiento debido a la COVID-19 en los servicios de VIH?

Haciendo click en el **SIGUIENTE** botón habrá terminado esta encuesta. Por favor compruebe sus respuestas antes de continuar.

¡Muchas gracias por su colaboración!
