# Supplementary material for: Mitigating the impact of COVID-19 on tuberculosis and HIV services: A cross-sectional survey of 669 health professionals in 64 low and middle-income countries
Source: PLoS One. 2021 Feb 2;16(2):e0244936. doi: 10.1371/journal.pone.0244936 (PMC7853462; doi:10.1371/journal.pone.0244936)
Supplement: S1 File — (ZIP) [file pone.0244936.s001.zip › Swahili Survey_FINAL.docx]

Identifying and mitigating impact of COVID-19 on TB and HIV programmes

Kutambua na kukabiliana na jinsi COVID-19 inavyodhulumu miradi ya magonjwa ya Kifua Kikuu (TB) na Ukimwi (HIV)

Information HABARI

- **We are conducting a short survey to understand ways in which TB and HIV services have been impacted by COVID-19 in low and middle income countries**

**Tunafanya uchunguzi fupi wa maono ili tupate kuelewa njia ambazo huduma za Kifua Kikuu na Ukimwi zimedhulumiwa na ugonjwa wa COVID-19 kwenye nchi masikini na zile za mapati ya wastani.**

- **The results will help to identify ways to protect and improve TB and HIV services**

**Matokeo yatasaidia kutambua njia za kulinda na kuimarisha huduma za kifua kikuu na ukimwi.**

- **This survey is for people who are involved in managing or delivering TB or HIV services (doctors, nurses, policymakers, health facility managers, community groups and researchers). The survey is not intended for patients.**

**Huu uchunguzi wa maono ni ya wale wanaohusika na kuendesha na kutoa huduma za kifua kikuu na ukimwi (madaktari, wauguzi, watoa maagizo, wakuu wa vituo vya afya, vikundi vya kijamii na watafiti). Haihusishi wagonjwa kamwe.**

- **You do not have to provide your name or any other details that will allow answers to be traced back to you. All information will be kept completely anonymous.**

**Si lazima upeane jina lako wala habari zingine za kibinafsi ambazo zinaweza kukubalia majibu yako kufuatiliwa hadi kukufikia. Habari zote zitawekwa bila majina.**

- **Depending on your area of work, you can answer questions about TB (approximately 15 minutes) or HIV (approximately 15 minutes) or both.**

**Kulingana na sehemu ipi unakofanya kazi, unaweza kuyajibu maswali kuhusu kifua kikuu (takriban dakika 15 -or– kumi na tano) au ukimwi (takriban dakika kumi na tano) au zote mbili.**

- **Once you start the survey you will need to complete it. You cannot save and come back, so please start the survey when you have enough time (15-30 minutes).**

**Mara unapoanza hii shughuli ya utoaji maono, utatakikana kuikamilisha. Hauwezi kuweka kando ili urudi kuikamilisha baadaye, kwa hivyo tafadhali ianze tu ikiwa una uakika kuwa unao mda wa kutosha (kati ya dakika 15 na 30 - thalathini).**

- **Please do not answer this survey more than once.**

**Tafadhali susijibu hii shughuli ya utoaji maono zaidi ya mara moja.**

**Detailed information about the study and your participation is available to download by clicking** [**here.**](https://docs.google.com/document/d/1L1MrsHnQUj1V72LJV2cYHe7oJlKA_OIAS1gkNWreOIA/edit)

**Habari zaidi kuhusu hii shughuli na kuhusika kwako inaweza kupatikana kwa kubonyeza** hapa.

- 1. **Consent to participate**

**Idhini ya kuhusishwa.**

By clicking the boxes below, I confirm that:

Kwa kubonyeza kwenye haya masunduku yafwatayo, ninahakikisha kwamba;

I have agreed to take part in the study

Nimekubali kuhusika na hii shughuli.

I have seen a copy of the information sheet (available by clicking the link above) that explains my role in this research. I understand its contents and agree to participate in this research.

Nimeiona kurasa la habari (kwa kubonyeza kiunganishi hapo juu) linaloeleza **kuhusika kw**angu kwenye utafiti huu.

I can withdraw from the survey at any point in time

Ninaweza kujiondoa kutoka kwa shughuli hii wakati wowote.

I will not have any financial benefits that result from the commercial development of this research

Sitakuwa na mapato ya kifedha yanayotokea kwa uimarishaji kibiashara kutokana na utafiti huu.

I consent to have the coded data made available for future research by putting it into a data repository

Napeana idhini ili majibu haya yaliyo chapishwa kwa ufiche yataweza kutumika kwa utafiti zaidi siku zijazo na kuhifadhiwa.

- 2. Thank you for your consent. If you provide text answers, do you agree for us to quote your statements (verbatim) in a report without identifying you?

Ahsante sana kwa kukubali. Ukijibu kwa maneno yako mwenyewe, unatukubalia kuyanukua (ulivyoyasema kikamili) kwenye ripoti bila kukutambulisha)

Yes

Ndiyo

No

La

- 3. What is your age?

Una umri gani?

- 4. What is your gender?

Ginsia yako ni ipi?

Female

Ya kike

Male

Ya kiume

Prefer not to answer

Naonelea nisijibu

Prefer to self describe:

Ningependelea kujieleza

- 5. Which of the following best describes the role you work in?

Ipi ifwatayo ineleza kamili kuhusika kwako kikazi?

Nurse providing care to patients

Muuguzi anaye wahudumia wagonjwa.

Doctor providing care to patients

Daktari anaye wahudumia wagonjwa.

Community healthcare worker

Mhudumu wa afya wa kijamii

Other healthcare provider

Mhudumu wa afya wa aina nyingine

Manager of healthcare facility or programme

Msimamizi (au meneja) wa kituo cha afya au mradi.

Researcher

Mtafiti

Other (please specify)

Wengineo (tafadhali eleza).

- 6. What type of organisation do you work in?

Je, unafanya kazi kwenye shirika la aina gani?

Public sector healthcare facility

Kituo cha afya cha umma?

**Private**, **for-profit** healthcare facility

Kituo cha afya cha kibinafsi na cha faida.

**Charity**/**non-profit** healthcare facility

Kituo cha afya kisicho cha faida au cha huduma za bure.

Government agency

Shirika la Serikali

Domestic non-governmental organization

Shirika kisilo la serikali la humu nchini

International non-governmental organization

Shirika lisilo la serikali la kimataifa

Funding agency

Watoaji msaada wa kifedha.

University or academic body

Chuo kikuu au cha kimasomo.

Other (please specify)

Zinginezo (tafadhali eleza)

- 7. Which country are you providing information about?

Unapeana habari kuhusu nchi gani?

- 8. Please select whether you would like to answer questions on TB, HIV or both

Tafadhalichagua ule ugonjwa ungependa kuyajibu maswali yake kati ya kifua kikuu na ukimwi, au yote mawili.

TB

Kifua kikuu

HIV

Ukimwi

Both

Yote mawili

Please answer the 9 short questions on TB. Thank you!

Tafadhali yajibu haya maswali tisa mafupi kuhusu kifua kikuu. Ahsante.

**You can select 'prefer not to answer' for any questions you want to skip.**

**Unaweza kuchagua ‘afadhali nisijibu’ kwa maswali yoyote ambayo ungependa yapitwe.**

- 9. Has it been harder for **healthcare providers to come to work** at TB healthcare facilities since COVID-19?

Je, imekuwa vigumu zaidi kwa wahudumu wa afya kufika kazini kwenye vituo vya afya vya kifua kikuu tangu mrupuko wa COVID-19?

No - same as before

La. Sawa tu na hapo awali.

Yes - it is slightly harder

Ndiyo. Kuna ugumu kidogo.

Yes - it is much harder

Ndiyo. Ni vigumu zaidi.

Yes – it is very difficult or impossible

Ndiyo. Ni vigumu sana au haiwezekani.

Don’t know

Sijui

Prefer not to answer

Afadhali nisijibu.

- 10. Has it been harder for **TB patients to access TB services** since COVID-19?

Je, wagonjwa wa kifua kikuu wamepata ugumu zaidi kuzifikia au kuzipata huduma zao tangu COVID-19?

No - same as before

La. Sawa tu na hapo awali.

Yes - it is slightly harder

Ndiyo. Kuna ugumu kidogo.

Yes - it is much harder

Ndiyo. Ni vigumu zaidi.

Yes – it is very difficult or impossible

Ndiyo. Ni vigumu sana au haiwezekani.

Don’t know

Sijui

Prefer not to answer

Afadhali nisijibu.

- 11. What do you think are the main **concerns or barriers for TB patients** to access healthcare since COVID-19? (select all that apply)

Unadhani ni vizuizi gani ambavyo au wasiwasi gani ambayo inawazuia wagonjwa wa kifua kikuu kupokea huduma za afya tangu COVID-19?

Physical distancing/lockdown rules

Sheria za kutokuwa karibu na wenngine au marufuku za kusafiri na kufungiwa.

Disruptions to transport

Ukosefu wa huduma za usafiri.

Reduced income/access to money to travel

Mapato pungufu au kutopata nauli ya kusafiri.

Fear of getting infected with COVID-19

Uoga kuwa labda watapata kuambukizwa ugonjwa wa COVID-19.

Closure of health facilities

Kufungwa kwa vituo vya afya.

Healthcare provider shortages

Upungufu wa wahudumu wa afya.

Longer waiting times

Kuongezeka kwa mda wa kuongojea huduma.

Unable to access a face mask

Kutoweza kupata barakoa.

There are NO concerns or barriers for TB patients

Hamna wasiwasi wala vizuizi kwa wagonjwa wa kifua kikuu.

Prefer not to answer

Afadhali nisijibu.

Other (please explain below)

Zinginezo (tafadhali jieleze hapo chini)

- 12. Since COVID-19, what **control measures have been implemented by the government** and how has this impacted TB health services? (examples: reduced transport, movement restrictions, etc)

Tangu COVID-19, je, ni hatua gani imetendwa na serikali, nah ii imehujumu kivipi huduma za kifua kikuu? (Kwa mfano, usafiri pungufu, kukatizwa matembezi, nk (abbrev. for ‘na kadhalika’))

- 13. Since COVID-19, are you aware of any changes to the way **TB healthcare facilities are operating**? (select all that apply)

Tangu COVID-19, una habari kuhusu mabadiliko yoyote vile vituo vya afya vya kifua kikuu vinaendeleza kazi?

No - same as before

La. Sawa tu na hapo awali.

Yes – physical distancing protocols for patients

Ndiyo – kutenganishwa kwa wagonjwa wasikaribiane.

Yes – masks or other protective equipment for healthcare providers

Ndiyo – barakoa na vifaa vya kukinga mwili kwa wahudumu wa afya.

Prefer not to answer/ don't know

Afadhali nisijibu.

/Sijui

Yes - Other, please explain below

Zinginezo (tafadhali jieleze hapo chini)

- 14. Have you experienced shortages of diagnostics or other challenges to provision of routine **diagnostic** **services** for TB since COVID-19?

Je, kumekuwa na upungufu wa vifaa vya uvumbuzi au kukumbwa na ugumu wa aina nyingine kuhusu uvumbuaji wa kifua kikuu tangu COVID-19?

No - same as before

La. Sawa tu na hapo awali.

Yes - it is slightly harder to provide diagnostic services

Ndiyo – ni vigumu kidogo kutoa hizo huduma za uvumbuaji.

Yes - it is much harder to provide diagnostic services

Ndiyo – ni vigumu zaidi kutoa hizo huduma.

Yes – it is very difficult or impossible to provide diagnostic services

Ndiyo – ni vigumu sana au haiwezekani kutoa hizo huduma.

Don’t know

Sijui

Prefer not to answer

Afadhali nisijibu.

Please use this space to provide more details about what has caused the change

Tafadhali tumia nafasi hii kupeana sababu zaidi zilizosababisha hayo mabadiliko.

- 15. Have you experienced shortages of medicines or other challenges to provision of standard **treatment** for TB patients since COVID-19?

Je, umepatwa na upungufu wa madawa au matatizo kwa kupeana matibabu ya kawaida kwa wagonjwa wa kifua kikuu tangu COVID-19?

No - same as before

La. Sawa tu na hapo awali.

Yes - it is slightly harder to provide TB treatment

Ndiyo – ni vigumu kidogo kutoa matibabu ya kifua kikuu.

Yes - it is much harder to provide TB treatment

Ndiyo – ni vigumu zaidi kutoa matibabu ya kifua kikuu.

Yes – it is very difficult or impossible to provide TB treatment

Ndiyo – ni vigumu sana au haiwezekani kutoa matibababu ya kifua kikuu.

Don’t know

Sijui

Prefer not to answer

Afadhali nisijibu.

Please use this space to provide more details, including challenges with ART for TB patients

Tafadhali tumia nafasi hii kupeana sababu zaidi zilizosababisha hayo mabadiliko.

pia matatizo na madawa za virusi kwa wagonjwa wa kifua kikuu.

- 16. Has it been harder for TB patients to access **non-medical support** such as food supplementation or counselling since COVID-19?

Je, imekuwa vigumu zaidi kwa wagonjwa wa kifua kikuu kupata usaidizi usiyokuwa wa kimatibabu kama vyakula au kupewa mawaidha tangu COVID-19?

No - same as before

La. Sawa tu na hapo awali.

Yes - it is slightly harder

Ndiyo. Kuna ugumu kidogo.

Yes - it is much harder

Ndiyo. Ni vigumu zaidi.

Yes – it is very difficult or impossible

Ndiyo. Ni vigumu sana au haiwezekani.

Not available in my country, region, or facility

Hazipatikani kwenye nchi yangu, mkoa wangu au kituo changu.

Don’t know

Sijui

Prefer not to answer

Afadhali nisijibu.

Please use this space to provide more details

Tafadhali tumia nafasi hii kupeana sababu zaidi zilizosababisha hayo mabadiliko.

1. What do you think can be done (or has already been done) to **minimize or avoid disruptions from** **COVID-19** to TB services?

Unadhani nini kinaweza kufanywa (au chenye kimeshafanywa) kupunguza au kukwepa dhulma za COVID-19 kwa huduma za kifua kikuu?

By clicking the **NEXT** button, you will end this survey. Please check your answers before continuing. Thank you for taking the time to answer this survey!

Kwa kubonyeza kifunguu kisemacho IJAYO, utakuwa umekamilisha shughuli ya hii utoaji wa maono. Tafadhali anagalia majibu yako kabla ya kuendelea.

Please answer the 9 short questions on HIV. Thank you!

**You can select 'prefer not to answer' for any questions you want to skip.**

- 18. Has it been harder for **healthcare providers to come to work** at HIV healthcare facilities since COVID-19?

Je, imekuwa vigumu zaidi kwa wahudumu wa afya kufika kazini kwenye vituo vya afya vya Ukimwi tangu mrupuko wa COVID-19?

No - same as before

La. Sawa tu na hapo awali.

Yes - it is slightly harder

Ndiyo. Kuna ugumu kidogo.

Yes - it is much harder

Ndiyo. Ni vigumu zaidi.

Yes – it is very difficult or impossible

Ndiyo. Ni vigumu sana au haiwezekani.

Don’t know

Sijui

Prefer not to answer

Afadhali nisijibu.

- 19. Has it been harder for **HIV patients to access HIV services** since COVID-19?

Je, wagonjwa wa Ukimwi wamepata ugumu zaidi kuzifikia au kuzipata huduma zao tangu COVID-19?

No - same as before

La. Sawa tu na hapo awali.

Yes - it is slightly harder

Ndiyo. Kuna ugumu kidogo.

Yes - it is much harder

Ndiyo. Ni vigumu zaidi.

Yes – it is very difficult or impossible

Ndiyo. Ni vigumu sana au haiwezekani.

Don’t know

Sijui

Prefer not to answer

Afadhali nisijibu.

- 20. What do you think are the main **concerns or barriers for HIV patients** to access healthcare since COVID-19? (select all that apply)

Unadhani ni vizuizi gani ambavyo au wasiwasi gani ambayo inawazuia wagonjwa wa Ukimwi kupokea huduma za afya tangu COVID-19?

Physical distancing/lockdown rules

Sheria za kutokuwa karibu na wenngine au marufuku za kusafiri na kufungiwa.

Disruptions to transport

Ukosefu wa huduma za usafiri.

Reduced income/access to money to travel

Mapato pungufu au kutopata nauli ya kusafiri.

Fear of getting infected with COVID-19

Uoga kuwa labda watapata kuambukizwa ugonjwa wa COVID-19.

Closure of health facilities

Kufungwa kwa vituo vya afya.

Healthcare provider shortages

Upungufu wa wahudumu wa afya.

Longer waiting times

Kuongezeka kwa mda wa kuongojea huduma.

Unable to access a face mask

Kutoweza kupata barakoa.

There are NO concerns or barriers for HIV patients

Hamna wasiwasi wala vizuizi kwa wagonjwa wa Ukimwi .

Prefer not to answer

Afadhali nisijibu.

Other (please explain below)

Zinginezo (tafadhali jieleze hapo chini)

- 21. Since COVID-19, what **control measures have been implemented by the government** and how has this impacted HIV health services? (examples: reduced transport, movement restrictions, etc)

Tangu COVID-19, je, ni hatua gani imetendwa na serikali, nah ii imehujumu kivipi huduma za Ukimwi ? (Kwa mfano, usafiri pungufu, kukatizwa matembezi, nk (abbrev. for ‘na kadhalika’))

- 22. Since COVID-19, are you aware of any changes to the way **HIV healthcare facilities are operating**? (select all that apply)

Tangu COVID-19, una habari kuhusu mabadiliko yoyote vile vituo vya afya vya Ukimwi vinaendeleza kazi?

No - same as before

La. Sawa tu na hapo awali.

Yes – physical distancing protocols for patients

Ndiyo – kutenganishwa kwa wagonjwa wasikaribiane.

Yes – masks or other protective equipment for healthcare providers

Ndiyo – barakoa na vifaa vya kukinga mwili kwa wahudumu wa afya.

Prefer not to answer/ don't know

Afadhali nisijibu.

/Sijui

Yes - Other, please explain below

Zinginezo (tafadhali jieleze hapo chini)

- 23. Have you experienced shortages of diagnostics or other challenges to provision of routine **diagnostic** **services** for HIV since COVID-19?

Je, kumekuwa na upungufu wa vifaa vya uvumbuzi au kukumbwa na ugumu wa aina nyingine kuhusu uvumbuaji wa Ukimwi tangu COVID-19?

No - same as before

La. Sawa tu na hapo awali.

Yes - it is slightly harder to provide diagnostic services

Ndiyo – ni vigumu kidogo kutoa hizo huduma za uvumbuaji.

Yes - it is much harder to provide diagnostic services

Ndiyo – ni vigumu zaidi kutoa hizo huduma.

Yes – it is very difficult or impossible to provide diagnostic services

Ndiyo – ni vigumu sana au haiwezekani kutoa hizo huduma.

Don’t know

Sijui

Prefer not to answer

Afadhali nisijibu.

Please use this space to provide more details about what has caused the change

Tafadhali tumia nafasi hii kupeana sababu zaidi zilizosababisha hayo mabadiliko.

- 24. Have you experienced shortages of medicines or other challenges to provision of standard **treatment** for HIV patients since COVID-19?

Je, umepatwa na upungufu wa madawa au matatizo kwa kupeana matibabu ya kawaida kwa wagonjwa wa Ukimwi tangu COVID-19?

No - same as before

La. Sawa tu na hapo awali.

Yes - it is slightly harder to provide HIV treatment

Ndiyo – ni vigumu kidogo kutoa matibabu ya Ukimwi .

Yes - it is much harder to provide HIV treatment

Ndiyo – ni vigumu zaidi kutoa matibabu ya Ukimwi .

Yes – it is very difficult or impossible to provide HIV treatment

Ndiyo – ni vigumu sana au haiwezekani kutoa matibababu ya Ukimwi .

Don’t know

Sijui

Prefer not to answer

Afadhali nisijibu.

Please use this space to provide more details, including challenges with ART for HIV patients

Tafadhali tumia nafasi hii kupeana sababu zaidi zilizosababisha hayo mabadiliko.

pia matatizo na madawa za virusi kwa wagonjwa wa Ukimwi .

- 25. Has it been harder for HIV patients to access **non-medical support** such as food supplementation or counselling since COVID-19?

Je, imekuwa vigumu zaidi kwa wagonjwa wa Ukimwi kupata usaidizi usiyokuwa wa kimatibabu kama vyakula au kupewa mawaidha tangu COVID-19?

No - same as before

La. Sawa tu na hapo awali.

Yes - it is slightly harder

Ndiyo. Kuna ugumu kidogo.

Yes - it is much harder

Ndiyo. Ni vigumu zaidi.

Yes – it is very difficult or impossible

Ndiyo. Ni vigumu sana au haiwezekani.

Not available in my country, region, or facility

Hazipatikani kwenye nchi yangu, mkoa wangu au kituo changu.

Don’t know

Sijui

Prefer not to answer

Afadhali nisijibu.

Please use this space to provide more details

Tafadhali tumia nafasi hii kupeana sababu zaidi zilizosababisha hayo mabadiliko.

26.What do you think can be done (or has already been done) to **minimize or avoid disruptions from** **COVID-19** to HIV services?

Unadhani nini kinaweza kufanywa (au chenye kimeshafanywa) kupunguza au kukwepa dhulma za COVID-19 kwa huduma za Ukimwi ?
