# Supplementary material for: Mitigating the impact of COVID-19 on tuberculosis and HIV services: A cross-sectional survey of 669 health professionals in 64 low and middle-income countries
Source: PLoS One. 2021 Feb 2;16(2):e0244936. doi: 10.1371/journal.pone.0244936 (PMC7853462; doi:10.1371/journal.pone.0244936)
Supplement: S1 File — (ZIP) [file pone.0244936.s001.zip › Urdu.docx]

**ٹی بی اورایچ آئی وی پروگرام پر کورونا وائرس کے اثرات کی نشاندہی اور**

**تخفیف**

معلومات Information

ہم ایک مختصر سروے کررہے ہیں جس کا مقصد کورونا وائرس کے ٹی بی اور ایچ آئی وی **پروگرام پر** ہونے والے اثرات کو سمجھنا ہے۔

اس سروے کے نتائج کی مدد سے ہم ٹی بی اور ایچ آئی وی خدمات کو محفوظ اور بہتر بناسکیں گے۔

یہ سروے ٹی بی اور ایچ آئی وی کی خدمات فراہم کرنے والے لوگوں کے لئے ہے۔ (ڈاکٹر ، نرسیں، حکمت عملی مرتب کرنے والے،صحت کی سہولیات فراہم کرنے والے اداروں کے مینیجر،( ہیلتھ فیسلیٹی مینیجرز)، کمیونٹی گروپس اور ریسرچر وغیرہ ) یہ سروے مریضوں کے لئے نہیں ہے۔

آپ کو اپنا نام یا دیگر کوائف ( جس کے ذریعے آپ تک رسائی ممکن ہو) دینے کی ضرورت نہیں ہے۔ تمام معلومات کو صیغہ راز میں رکھا جائےگا۔

آپ اپنے کام کی نوعیت کے حساب سے ٹی بی ( تقریبا پندرہ منٹ ) یا ایچ آئی وی ( تقریبا پندرہ منٹ ) یا دونوں کے سوالات کے جوابات دے سکتے ہیں

ایک بار جب آپ سروے شروع کردیں تو آپ کو اسے مکمل کرنے کی ضرورت ہوگی۔ آپ سوالات کو مکمل کیے بغیر واپس نہیں آسکتے

، لہذا جب آپ کے پاس کافی وقت ہوگا تو براہ کرم سروے شروع کریں۔

اس سروے کاایک سے زائد مرتبہ جواب دینے سے پرہیز کریں۔

اس سروے کے حوالے سے تفصیلی معلومات اور آپ کی اس میں شرکت کے حوالے سے دوسری معلومات ڈاؤ ن لوڈکرنے کے لئے مندرجہ ذیل لنک کو دبائیں۔

(۱) سروے میں شرکت کی رضامندی

نیچے دیئے گئے بکس کو کلک کریں ۔ میں تصدیق کرتا ہوں /کرتی ہوں۔

🟈 میں نے اس مطالع میں شرکت کے لئے رضامندی ظاہر کی ہے۔

- میں نے معلوماتی پرچہ کی کاپی دیکھی ہے۔ جو اس تحقیق کے حوالے سے میرے کردار کی وضاحت کرتی ہے۔
- مجھے اس سروے سے کسی بھی وقت علیحدگی کا اختیار ہے۔
- اس تحقیق کے تجارتی اثرات سے مجھے کوئی مالی فوائد حاصل نہیں ہوں گے۔
- میں اجازت دیتا /دیتی ہوں کہ یہ کوڈ ڈ ڈیٹا مستقبل کی تحقیق کے لئے ڈیٹا ریپوزٹری (ذخیرہ ) میں دستیاب ہوگا۔

(۲) آپ کی رضامندی کا شکریہ ! اگر آپ اجازت دیں تو(آپ کی شناخت کو ظاہر کئے بغیر) آپ کے جوابات کے متن کا حوالہ رپورٹ میں دے سکتے ہیں۔

(۳) عمر

(۴) جنس

مرد

عورت

جواب نہیں دینا چاہتی /چاہتا

(۵) مندر جہ ذیل میں سے آپ کے کا م /شعبہ کی نوعیت کی وضاحت کون سے کردار سے ہے؟

✔ نرس (مریضوں کی دیکھ بھال)

✔ ڈاکٹر

✔ کمیونٹی ہیلتھ ورکر

کو ئی اور ہیلتھ ورکر

✔ ہیلتھ فیسلیٹی مینیجر

✔ ریسرچر

✔ کو ئی اور (ضرور وضاحت کریں)

(۶) آپ کس قسم کی تنظیم ( آرگنائزیشن ) میں کام کرتے /کرتی ہیں۔

✔ گورنمنٹ سیکٹر ادارہ براۓ صحت

✔ پرائیوٹ سیکٹر ادارہ براۓ صحت

✔ خیراتی ادارہ براۓ صحت

✔ سرکاری ایجنسی

✔ صوبائی /ضلعی این جی او

✔ بین الاقوامی این جی او

✔ فنڈنگ ایجنسی

✔ تعلیمی ادارہ

✔ کوئی اور وضاحت کریں۔

(۷) آپ کس ملک کے حوالے سے معلومات دے رہے /رہی ہیں۔

(۸) مندر جہ ذیل میں سے کوئی ایک آپشن منتخب کریں جس کے بارے میں آپ جواب دینا چاہیں گے۔

ٹی بی

ایچ آئی وی

دونوں

ٹی بی کے حوالے سے نو چھوٹے سوالات کے جواب دیجئے ۔ شکریہ

اگر آپ کسی سوال کا جواب نہیں دینا چاہتے /چاہتی ہوں تو اسے نظر انداز کرسکتے ہیں۔

(۹) کیا کورونا وائرس کی وجہ سے ہیلتھ کیئر پرووائڈر کا ٹی بی ادارہ براۓ صحت پحنچنا پہلے سے مشکل ہو گیا ہے ؟

جی نہیں ۔ پہلے کی طرح ہی ہیں

جی ہاں ۔ تھوڑی مشکل ہوگئی ہے۔

جی ہاں ۔ کافی مشکلات درپیش ہیں۔

جی ہاں ۔ بہت مشکل یا ناممکن ہے۔

نہیں معلوم

جواب نہیں دینا چاہتا /چاہتی

(۱۰) کیا کورونا وائرس کی وجہ سے ٹی بی کے مریضوں کو ٹی بی خدمات کی رسائی میں مشکلات کا سامنا ہے؟

جی نہیں ۔ پہلے کی طرح ہی ہیں

جی ہاں ۔ تھوڑی مشکل ہوگئی ہے۔

جی ہاں ۔ کافی مشکلات درپیش ہیں۔

جی ہاں ۔ بہت مشکل یا ناممکن ہے۔

نہیں معلوم

جواب نہیں دینا چاہتا /چاہتی

(۱۱) آپ کے مطابق کورونا وائرس کی وجہ سے ٹی بی کے مریضوں کو کونسی رکاوٹیں یا خدشات کا سامنا ہے جس کے باعث وہ صحت کی سہولیات تک رسائی حاصل نہیں کرپارہے ہیں ؟ ایک سے زائد آپشن کو منتخب کرسکتے ہیں۔

✔ لاک ڈاون / لوگوں سے فاصلہ رکھنا

ٹرانسپورٹ کی بندش

کم آمدنی / سفر کے لۓ رقم کا نہ ہونا

کورونا وائرس انفیکشن لگ جانے کا ڈر

صحت کی سہولیات کا بند ہونا

صحت کی سہولیات دینے والے افراد کی قلت

طویل انتظار

فیس ماسک کی عدم دستیابی

ٹی بی کے مریضوں کوکوئی رکاوٹ یا خدشات کا سامنا نہیں ہے ۔

جوا ب نہیں دینا چاہتا/چاہتی

کوئی اور وضاحت کریں

(۱۲( جب سے کورونا وائرس پھیلا ہے ، حکومت نے کن کن کنٹرول اقدامات کو نافذ کیا ہے اور اس سے ٹی بی کی سہولیات کیسے متاثر ہوئ ہیں۔ (مثال کے طور پر نقل و حرکت کی پابندی، وغیرہ)؟

(۱۳) جب سے کورونا وائرس پھیلا ہے ، کیا آپ کسی ایسی تبدیلی سے واقف ہیں جو ٹی بی کلینک میں آ پ نے نوٹس کی ہو؟

جی نہیں ۔ پہلے کی طرح ہی ہیں۔

جی ہاں ۔ مریضوں کے درمیان فاصلہ اور دوری کے پروٹوکول کا مشاہدہ کیا۔

جی ہاں ۔ ہیلتھ کئیر پرووائڈر کے لئے ماسک اور پی پی ای کی فراہمی

جواب نہیں/ معلوم نہیں

کوئی اور وضاحت کریں

(۱۴( کورونا وائرس کے دوران کیا آپ کو ٹی بی کی تشخیصی خدمات میں رکاوٹ یا قلت یا کوئی اور چیلنچ کا سامنا کرنا پڑا۔

جی نہیں۔ پہلے کی طرح ہی ہے۔

جی ہاں۔ تھوڑی مشکلات درپیش ہیں۔

جی ہاں ۔ کافی مشکلات درپیش آئی

جی ہاں ۔ تشخیصی خدمات فراہم کرنا بہت ہی مشکل یا نا مممکن ہے۔

معلوم نہیں ۔

جواب نہیں دینا چاہتا/چاہتی

نیچے دیئے گئے خانے میں تفصیلی جواب ضرور تحریر کریں۔

✔ کہ آپ کو کس قسم کی مشکلات کا سامنا کرنا پڑا۔

(۱۵) کیا آپ کو کورونا وائرس کی وبا کے دوران ٹی بی کے علاج کی ادویات کی قلت یا دوسری کسی اور مشکلات کا سامنا کرنا پڑا۔

جی نہیں۔ پہلے جیسی صورت حال ہے۔

جی ہاں۔ تھوڑی مشکلات درپیش ہیں

جی ہاں ۔ کافی مشکلات درپیش آئی مریضوں کے علاج میں

جی ہاں ۔ ٹی بی کے علاج کے حوالے سے بے تحاشا مشکلات دریپش ہیں

معلوم نہیں

جواب نہیں دینا چاہتا/ چاہتی

نیچے دیئے گئے خانے میں مزید تفصیلات بیان کریں

(۱۶) کیا کورونا وائرس کے دوران مریضوں کے لئے سہولیات غیر طبی اور مشاورت جیسی سہولیات تک رسائی مشکل عمل بن گئی ۔

جی نہیں ۔ پہلے جیسی سہولت ہے۔

جی ہاں۔ تھوڑی مشکلات درپیش ہیں

جی ہاں ۔ کافی مشکل ہے۔

جی ہاں ۔ بے تحاشا مشکل اور ناممکن لے۔

میرے ملک /علاقے میں یہ سہولت میسر نہیں ہے۔

معلوم نہیں

جواب نہیں دینا چاہتا / چاہتی

نیچے دیئے گئے خانے میں مزید تفصیلات بیان کریں۔

(۱۷) آپ کے خیال میں کون سے اقدامات کرنے چاہیئں ( یا اقدامات لئے جاچکے ہیں ) تاکہ ٹی بی کی خدمات میں تعطل کو کم کیا جاسکے ۔

آپ اب **نیکسٹ** بٹن دباکر سروے کو ختم کرسکتے ہیں۔ آگے بڑھنے سے پہلے جوابا ت کو دوبارہ چیک کرلیں۔ آپ کے قیمتی وقت کا بہت شکریہ !

ایچ آئی وی کے حوالے سے نو چھوٹے سوالات کے جواب دیجئے ۔ شکریہ

اگر آپ کسی سوال کا جواب نہیں دینا چاہتے /چاہتی ہوں تو اسے نظر انداز کرسکتے ہیں۔

(۱۸) کیا کورونا وائرس کی وجہ سے ہیلتھ کیئر پرووائڈر کا ایچ آئی وی ادارہ براۓ صحت پحنچنا پہلے سے مشکل ہو گیا ہے ؟

جی نہیں ۔ پہلے کی طرح ہی ہیں

جی ہاں ۔ تھوڑی مشکل ہوگئی ہے۔

جی ہاں ۔ کافی مشکلات درپیش ہیں۔

جی ہاں ۔ بہت مشکل یا ناممکن ہے۔

نہیں معلوم

جواب نہیں دینا چاہتا /چاہتی

(۹ ۱) کیا کورونا وائرس کی وجہ سے ایچ آئی وی کے مریضوں کو ایچ آئی وی خدمات کی رسائی میں مشکلات کا سامنا ہے؟

جی نہیں ۔ پہلے کی طرح ہی ہیں

جی ہاں ۔ تھوڑی مشکل ہوگئی ہے۔

جی ہاں ۔ کافی مشکلات درپیش ہیں۔

جی ہاں ۔ بہت مشکل یا ناممکن ہے۔

نہیں معلوم

جواب نہیں دینا چاہتا /چاہتی

(۰ ۲) آپ کے مطابق کورونا وائرس کی وجہ سے ایچ آئی وی کے مریضوں کو کونسی رکاوٹیں یا خدشات کا سامنا ہے جس کے باعث وہ صحت کی سہولیات تک رسائی حاصل نہیں کرپارہے ہیں ؟ ایک سے زائد آپشن کو منتخب کرسکتے ہیں۔

✔ لاک ڈاون / لوگوں سے فاصلہ رکھنا

ٹرانسپورٹ کی بندش

کم آمدنی / سفر کے لۓ رقم کا نہ ہونا

کورونا وائرس انفیکشن لگ جانے کا ڈر

صحت کی سہولیات کا بند ہونا

صحت کی سہولیات دینے والے افراد کی قلت

طویل انتظار

فیس ماسک کی عدم دستیابی

ایچ آئی وی کے مریضوں کوکوئی رکاوٹ یا خدشات کا سامنا نہیں ہے ۔

جوا ب نہیں دینا چاہتا/چاہتی

کوئی اور وضاحت کریں

(۲۱ ( جب سے کورونا وائرس پھیلا ہے ، حکومت نے کن کن کنٹرول اقدامات کو نافذ کیا ہے اور اس سے ایچ آئی وی کی سہولیات کیسے متاثر ہوئ ہیں۔ (مثال کے طور پر نقل و حرکت کی پابندی، وغیرہ)؟

(۲۲) جب سے کورونا وائرس پھیلا ہے ، کیا آپ کسی ایسی تبدیلی سے واقف ہیں جو ایچ آئی وی کلینک میں آ پ نے نوٹس کی ہو؟

جی نہیں ۔ پہلے کی طرح ہی ہیں۔

جی ہاں ۔ مریضوں کے درمیان فاصلہ اور دوری کے پروٹوکول کا مشاہدہ کیا۔

جی ہاں ۔ ہیلتھ کئیر پرووائڈر کے لئے ماسک اور پی پی ای کی فراہمی

جواب نہیں/ معلوم نہیں

کوئی اور وضاحت کریں

(۲۳ ( کورونا وائرس کے دوران کیا آپ کو ایچ آئی وی کی تشخیصی خدمات میں رکاوٹ یا قلت یا کوئی اور چیلنچ کا سامنا کرنا پڑا۔

جی نہیں۔ پہلے کی طرح ہی ہے۔

جی ہاں۔ تھوڑی مشکلات درپیش ہیں۔

جی ہاں ۔ کافی مشکلات درپیش آئی

جی ہاں ۔ تشخیصی خدمات فراہم کرنا بہت ہی مشکل یا نا مممکن ہے۔

معلوم نہیں ۔

جواب نہیں دینا چاہتا/چاہتی

نیچے دیئے گئے خانے میں تفصیلی جواب ضرور تحریری کریں۔

کہ آپ کو کس قسم کی مشکلات کا سامنا کرنا پڑا۔

(۲۴) کیا آپ کو کورونا وائرس کی وبا کے دوران ایچ آئی وی کے علاج کی ادویات کی قلت یا دوسرے کسی اور مشکلات کا سامنا کرنا پڑا۔

جی نہیں۔ پہلے جیسی صورت حال ہے۔

جی ہاں۔ تھوڑی مشکلات درپیش ہیں

جی ہاں ۔ کافی مشکلات درپیش آئی مریضوں کے علاج میں

جی ہاں ۔ ایچ آئی وی کے علاج کے حوالے سے بے تحاشا مشکلات دریپش ہیں

معلوم نہیں

جواب نہیں دینا چاہتا/ چاہتی

نیچے دیئے گئے خانے میں مزید تفصیلات بیان کریں

(۵۲) کیا کورونا وائرس کے دوران مریضوں کے لئے سہولیات غیر طبی اور مشاورت جیسی سہولیات تک رسائی مشکل عمل بن گئی ۔

جی نہیں ۔ پہلے جیسی سہولت ہے۔

جی ہاں۔ تھوڑی مشکلات درپیش ہیں

جی ہاں ۔ کافی مشکل ہے۔

جی ہاں ۔ بے تحاشا مشکل اور ناممکن لے۔

میرے ملک /علاقے میں یہ سہولت میسر نہیں ہے۔

معلوم نہیں

جواب نہیں دینا چاہتا / چاہتی

نیچے دیئے گئے خانے میں مزید تفصیلات بیان کریں۔

(۶۲) آپ کے خیال میں کون سے اقدامات کرنے چاہیئں ( یا اقدامات لئے جاچکے ہیں )

تاکہ ایچ آئی وی کی خدمات میں تعطل کو کم کیا جاسکے

آپ اب **نیکسٹ** بٹن دباکر سروے کو ختم کرسکتے ہیں۔ آگے بڑھنے سے پہلے جوابا ت کو دوبارہ چیک کرلیں۔ آپ کے قیمتی وقت کا بہت شکریہ !

پہلے ٹی بی کے حوالے سے نو چھوٹے سوالات کے جواب دیجئے

اس کے بعد ایچ آئی وی کے حوالے سے نو چھوٹے سوالات کے جواب دیجئے

اگر آپ کسی سوال کا جواب نہیں دینا چاہتے /چاہتی ہوں تو اسے نظر انداز کرسکتے ہیں
