# Supplementary material for: Mitigating the impact of COVID-19 on tuberculosis and HIV services: A cross-sectional survey of 669 health professionals in 64 low and middle-income countries
Source: PLoS One. 2021 Feb 2;16(2):e0244936. doi: 10.1371/journal.pone.0244936 (PMC7853462; doi:10.1371/journal.pone.0244936)
Supplement: S2 File — (DOCX) [file pone.0244936.s002.docx]

**Identifying and mitigating impact of COVID-19 on TB and HIV programmes**

We are conducting a short survey to understand ways in which HIV and TB services have been impacted by COVID-19 in low and middle income countries in order to identify ways in which disruptions can be minimised.

The survey will take approximately 15 minutes to complete if you answer questions about either TB or HIV or approximately 30 minutes if you answer questions about both TB and HIV.

Detailed information about the study and your participation is available to download by clicking here.

Please do not answer this survey more than once.

**Consent to participate**

By clicking on the box below, I confirm that:

1. I have agreed to take part in the study

2. I have seen a copy of the information sheet (available by clicking the link above) that explains my role in this research. I understand its contents and agree to participate in this research.

3. I can withdraw from the survey at any point in time

4. I will not have any financial benefits that result from the commercial development of this research

5. I consent to have the coded data made available for future research by putting it into a data repository

6. I agree for verbatim quotes to be used without identifying me

**Demographic Questions**

1. **Age**
   1. 18-24
   2. 25-34
   3. 35-44
   4. 45-54
   5. 5-64
   6. 65-74
   7. 75 +
2. **Gender**
   1. Male
   2. Female
3. **Which position do you work in?**

Drop down menu to select from

1. **Which country are you working in?**

Drop down menu to select from

*Please select whether you would like to answer questions on TB, HIV or both [once the participant clicks, they will be directed to the appropriate series of questionnaire]*

*TB*

*HIV*

*Both*

**Disruptions to TB Care Questions**

**To what extent has the ability of health providers to access healthcare facilities been disrupted by COVID-19 in your country?**

No change, same as before

Small impact

Moderate impact

High impact

**To what extent has physical access of patients to TB healthcare facilities been disrupted by COVID-19 in your country?**

No change, same as before

Small impact

Moderate impact

High impact

[Please use this space to provide more details: open text answer]

1. **To what extent have routine diagnostic services for TB been disrupted?**

No change, same as before

Small impact

Moderate impact

High impact

[Please use this space to provide more details: open text answer]

1. **To what extent is there disruption to provision of usual treatment for TB patients?**

No change, same as before

Small impact

Moderate impact

High impact

[Please use this space to provide more details: open text answer]

1. **To what extent has access to social care (non-medical support such as food supplementation, or counseling) been affected?**

No change, same as before

Small impact

Moderate impact

High impact

[Please use this space to provide more details: open text answer]

1. **What do you think are the main concerns or challenges of TB?**

[Free text answer]

1. **What do you think are the main concerns or challenges of TB healthcare providers?**

[Free text answer]

1. **What measures do you think could be taken to minimize or avoid disruptions from COVID-19 to TB services?**

[Free text answer]

**Disruptions to HIV Care Questions**

**To what extent has the ability of health providers to access healthcare facilities been disrupted by COVID-19 in your country?**

No change, same as before

Small impact

Moderate impact

High impact

**To what extent has physical access of patients to HIV healthcare facilities been disrupted by COVID-19 in your country?**

No change, same as before

Small impact

Moderate impact

High impact

[Please use this space to provide more details: open text answer]

1. **To what extent have routine diagnostic services for HIV been disrupted?**

No change, same as before

Small impact

Moderate impact

High impact

[Please use this space to provide more details: open text answer]

1. **To what extent is there disruption to provision of usual treatment for HIV patients?**

No change, same as before

Small impact

Moderate impact

High impact

[Please use this space to provide more details: open text answer]

1. **To what extent has access to social care (non-medical support) been affected?**

No change, same as before

Small impact

Moderate impact

High impact

[Please use this space to provide more details: open text answer]

1. **What do you think are the main concerns or challenges of HIV patients?**

[Free text answer]

1. **What do you think are the main concerns or challenges of HIV healthcare providers?**

[Free text answer]

1. **What measures do you think could be taken to minimize or avoid disruptions from COVID-19?**

[Free text answer]
