# Supplementary material for: Mitigating the impact of COVID-19 on tuberculosis and HIV services: A cross-sectional survey of 669 health professionals in 64 low and middle-income countries
Source: PLoS One. 2021 Feb 2;16(2):e0244936. doi: 10.1371/journal.pone.0244936 (PMC7853462; doi:10.1371/journal.pone.0244936)
Supplement: S3 File — (DOCX) [file pone.0244936.s003.DOCX]

**Identifying and mitigating impact of COVID-19 on TB and HIV programmes**

- We are conducting a short survey to understand ways in which TB and HIV services have been impacted by COVID-19 in low and middle income countries
- The results will help to identify ways to protect and improve TB and HIV services
- This survey is for people who are involved in managing or delivering TB or HIV services (doctors, nurses, policymakers, health facility managers, community groups and researchers). The survey is not intended for patients.
- You do not have to provide your name or any other details that will allow answers to be traced back to you. All information will be kept completely anonymous.
- Depending on your area of work, you can answer questions about TB (approx 15 minutes) or HIV (approx 15 minutes) or both.
- Once you start the survey you will need to complete it. You cannot save and come back, so please start the survey when you have enough time (15-30 minutes)
- Please do not answer this survey more than once.

Detailed information about the study and your participation is available to download by clicking here.

1. **Consent to participate**

By clicking on the box below, I confirm that:

a. I have agreed to take part in the study

b. I have seen a copy of the information sheet (available by clicking the link above) that explains my role in this research. I understand its contents and agree to participate in this research.

c. I can withdraw from the survey at any point in time

d. I will not have any financial benefits that result from the commercial development of this research

e. I consent to have the coded data made available for future research by putting it into a data repository

1. Thank you for consenting! Could you confirm whether you agree for verbatim quotes to be used without identifying you?

Yes

No

**Demographic Questions**

1. **Age**
   1. 18-24
   2. 25-34
   3. 35-44
   4. 45-54
   5. 55-64
   6. 65+
   7. Prefer not to answer
2. **Which best describes your gender?**
   1. Female
   2. Male
   3. Prefer not to say
   4. Prefer to self-describe:
3. **Which of the following best describes the role you work in?**
   1. Nurse providing care to patients
   2. Doctor providing care to patients
   3. Community healthcare worker
   4. Other healthcare provider
   5. Manager of healthcare facility or programme
   6. Researcher
   7. Other: please specify
4. **What type of organisation do you work in?**
   1. Government healthcare facility
   2. Private, for-profit healthcare facility
   3. Private, non-profit healthcare facility
   4. Government agency
   5. Domestic non-governmental organisation
   6. International non-governmental organisation
   7. Funding agency
   8. University or academic body
   9. Other: please specify
5. **Which low or middle income country are you providing information about?**

Drop down menu to select from

1. *Please select whether you would like to answer questions on TB, HIV or both [once the participant clicks, they will be directed to the appropriate series of questionnaire]*

*TB*

*HIV*

*Both*

**TB Care Questions**

**Has it been harder for HEALTHCARE PROVIDERS to come to work at TB healthcare facilities since the COVID-19 outbreak began?**

No - same as before

Yes - it is slightly harder

Yes - it is much harder

Yes – it is very difficult or impossible

Don’t know

Prefer not to answer

**Has it been harder for TB PATIENTS to reach TB services since the COVID-19 outbreak began?**

No - same as before

Yes - it is slightly harder

Yes - it is much harder

Yes – it is very difficult or impossible

Don’t know

Prefer not to answer

1. **What do you think are the main concerns or BARRIERS FOR TB PATIENTS to access healthcare since COVID-19?**
   1. Physical distancing/lockdown rules
   2. Disruptions to transport
   3. Reduced income/access to money to travel
   4. Fear of getting infected with COVID-19
   5. Closure of health facilities
   6. Healthcare provider shortages
   7. Longer waiting times
   8. Other

If other please specify

1. **Since COVID-19, what are the main changes affecting TB patients or healthcare providers coming to health services? [transport issues, fear of infection etc]**

[Free text answer]

1. **Since COVID-19, are you aware of any changes to the way TB HEALTHCARE FACILITIES ARE OPERATING?**

No - same as before

Yes – physical distancing protocols for patients

Yes – masks or other protective equipment for healthcare providers

Yes – Other

If Other please specify

1. **Have you experienced shortages of diagnostics or other challenges to provision of routine DIAGNOSTIC SERVICES for TB since the COVID-19 outbreak began?**

No - same as before

Yes - it is slightly harder to provide diagnostic services

Yes - it is much harder to provide diagnostic services

Yes – it is very difficult or impossible to provide diagnostic services

Don’t know

Prefer not to answer

[Please use this space to provide more details about what has caused the change: Free text answer]

1. **Have you experienced shortages of medicines or other challenges to provision of standard TREATMENT for TB patients since the COVID-19 outbreak began?**

No - same as before

Yes - it is slightly harder to provide TB treatment

Yes - it is much harder to provide TB treatment

Yes – it is very difficult or impossible to provide TB treatment

Don’t know

Prefer not to answer

[Please use this space to provide more details about what has caused the change: Free text answer]

1. **Has it been harder for TB patients to access to NON-MEDICAL SUPPORT such as food supplementation or counselling since the COVID-19 outbreak began?**

No - same as before

Yes - it is slightly harder

Yes - it is much harder

Yes – it is very difficult or impossible

Not available in my country

Don’t know

Prefer not to answer

[Please use this space to provide more details: Free text answer]

1. **What measures do you think could be taken to minimize or avoid disruptions from COVID-19 to TB services now and in the near future?**

[Free text answer]

**HIV Care Questions**

**Has it been harder for healthcare providers to reach HIV healthcare facilities they work at since the COVID-19 outbreak began?**

No - same as before

Yes - it is slightly harder

Yes - it is much harder

Yes – it is very difficult or impossible

Don’t know

Prefer not to answer

**Has it been harder for HIV patients to reach HIV services since the COVID-19 outbreak began?**

No - same as before

Yes - it is slightly harder

Yes - it is much harder

Yes – it is very difficult or impossible

Don’t know

Prefer not to answer

1. **In your opinion, what are the main factors impacting movement of HIV patients or healthcare providers to health services?**

[Free text answer]

1. **Has it been harder to provide routine diagnostic services for HIV since the COVID-19 outbreak began?**

No - same as before

Yes - it is slightly harder

Yes - it is much harder

Yes – it is very difficult or impossible

Don’t know

Prefer not to answer

[Please use this space to provide more details about what has caused the change: Free text answer]

1. **Has it been harder to provide HIV treatment for HIV patients since the COVID-19 outbreak began?**

No - same as before

Yes - it is slightly harder

Yes - it is much harder

Yes – it is very difficult or impossible

Don’t know

Prefer not to answer

[Please use this space to provide more details about what has caused the change: Free text answer]

1. **Has it been harder for HIV patients to access to non-medical support such as food supplementation or counselling since the COVID-19 outbreak began?**

No - same as before

Yes - it is slightly harder

Yes - it is much harder

Yes – it is very difficult or impossible

Not available in my country

Don’t know

Prefer not to answer

[Please use this space to provide more details: Free text answer]

1. **What do you think are the main concerns or challenges for:**
   1. **HIV patients?** [Free text answer]
   2. **HIV healthcare providers?** [Free text answer]
2. **What measures do you think could be taken to minimize or avoid disruptions from COVID-19 to HIV services now and in the near future?**

[Free text answer]

**TB and HIV Questions**

**Has it been harder for healthcare providers to reach TB healthcare facilities they work at since the COVID-19 outbreak began?**

No - same as before

Yes - it is slightly harder

Yes - it is much harder

Yes – it is very difficult or impossible

Don’t know

Prefer not to answer

**Has it been harder for TB patients to reach TB services since the COVID-19 outbreak began?**

No - same as before

Yes - it is slightly harder

Yes - it is much harder

Yes – it is very difficult or impossible

Don’t know

Prefer not to answer

1. **In your opinion, what are the main factors impacting movement of TB patients or healthcare providers to health services?**

[Free text answer]

1. **Has it been harder to provide routine diagnostic services for TB since the COVID-19 outbreak began?**

No - same as before

Yes - it is slightly harder

Yes - it is much harder

Yes – it is very difficult or impossible

Don’t know

Prefer not to answer

[Please use this space to provide more details about what has caused the change: Free text answer]

1. **Has it been harder to provide TB treatment for TB patients since the COVID-19 outbreak began?**

No - same as before

Yes - it is slightly harder

Yes - it is much harder

Yes – it is very difficult or impossible

Don’t know

Prefer not to answer

[Please use this space to provide more details about what has caused the change: Free text answer]

1. **Has it been harder for TB patients to access to non-medical support such as food supplementation or counselling since the COVID-19 outbreak began?**

No - same as before

Yes - it is slightly harder

Yes - it is much harder

Yes – it is very difficult or impossible

Not available in my country

Don’t know

Prefer not to answer

[Please use this space to provide more details: Free text answer]

1. **What do you think are the main concerns or challenges for:**
   1. **TB patients?** [Free text answer]
   2. **TB healthcare providers?** [Free text answer]
2. **What measures do you think could be taken to minimize or avoid disruptions from COVID-19 to TB services now and in the near future?**

[Free text answer]

**Has it been harder for healthcare providers to reach HIV healthcare facilities they work at since the COVID-19 outbreak began?**

No - same as before

Yes - it is slightly harder

Yes - it is much harder

Yes – it is very difficult or impossible

Don’t know

Prefer not to answer

**Has it been harder for HIV patients to reach HIV services since the COVID-19 outbreak began?**

No - same as before

Yes - it is slightly harder

Yes - it is much harder

Yes – it is very difficult or impossible

Don’t know

Prefer not to answer

1. **In your opinion, what are the main factors impacting movement of HIV patients or healthcare providers to health services?**

[Free text answer]

1. **Has it been harder to provide routine diagnostic services for HIV since the COVID-19 outbreak began?**

No - same as before

Yes - it is slightly harder

Yes - it is much harder

Yes – it is very difficult or impossible

Don’t know

Prefer not to answer

[Please use this space to provide more details about what has caused the change: Free text answer]

1. **Has it been harder to provide HIV treatment for HIV patients since the COVID-19 outbreak began?**

No - same as before

Yes - it is slightly harder

Yes - it is much harder

Yes – it is very difficult or impossible

Don’t know

Prefer not to answer

[Please use this space to provide more details about what has caused the change: Free text answer]

1. **Has it been harder for HIV patients to access to non-medical support such as food supplementation or counselling since the COVID-19 outbreak began?**

No - same as before

Yes - it is slightly harder

Yes - it is much harder

Yes – it is very difficult or impossible

Not available in my country

Don’t know

Prefer not to answer

[Please use this space to provide more details: Free text answer]

1. **What do you think are the main concerns or challenges for:**
   1. **HIV patients?** [Free text answer]
   2. **HIV healthcare providers?** [Free text answer]
2. **What measures do you think could be taken to minimize or avoid disruptions from COVID-19 to HIV services now and in the near future?**

[Free text answer]
