# Supplementary material for: Mitigating the impact of COVID-19 on tuberculosis and HIV services: A cross-sectional survey of 669 health professionals in 64 low and middle-income countries
Source: PLoS One. 2021 Feb 2;16(2):e0244936. doi: 10.1371/journal.pone.0244936 (PMC7853462; doi:10.1371/journal.pone.0244936)
Supplement: S1 File — (ZIP) [file pone.0244936.s001.zip › Russian.doc]

Выявление и минимизация негативного влияния COVID-19 на программы ТБ и ВИЧ

Информация

 **Мы проводим короткое исследование, чтобы разобраться, как именно COVID-19 сказался на ТБ- и ВИЧ-услугах в странах с низким и средним уровнем доходов**

 **Результаты помогут нам определить способы защиты и улучшения услуг в сфере ТБ и ВИЧ**

 **В опросе принимают участие люди, занимающиеся организацией или оказанием услуг в сфере ТБ или ВИЧ (врачи, медсестры, лица, определяющие политику, руководители учреждений здравоохранения, представители сообществ и исследователи). Опрос не предназначен для пациентов.**

 **Вам не нужно указывать свое имя или другие данные, которые позволят отследить ваши ответы. Вся информация будет полностью анонимной.**

 **В зависимости от сферы работы, вы можете отвечать на вопросы о ТБ (около 15 минут) или ВИЧ (около 15 минут), или же на вопросы из обеих этих категорий.**

 **Начав проходить опрос, вам придется пройти его до конца. Нельзя сохранить ответы, чтобы вернуться к опросу позже. Поэтому, пожалуйста, начинайте проходить опрос тогда, когда у вас будет достаточно времени (15-30 минут).**

 **Не следует проходить опрос больше одного раза.**

**Подробные сведения об исследовании и вашем участии в нем можно получить, нажав** [**сюда.**](https://docs.google.com/document/d/1L1MrsHnQUj1V72LJV2cYHe7oJlKA_OIAS1gkNWreOIA/edit)

- 1. **Согласие на участие**

Отмечая приведенные ниже пункты, я подтверждаю, что:

Я согласен(-на) принять участие в исследовании

Я читал(-а) информационный листок (доступный по приведенной выше ссылке), в котором объясняется моя роль в этом исследовании. Я понимаю его содержание и согласен(-на) принять участие в данном исследовании.

Я могу отказаться от участия в опросе в любой момент

Я не получу никаких финансовых выгод от коммерческого использования этого исследования

Я согласен(-на) на то, чтобы закодированные данные были помещены в хранилище с возможностью использования их в будущих исследованиях

- 2. Благодарим за ваше согласие. Согласны ли вы, чтобы мы цитировали в отчете ваши слова (без искажений) из ответов на открытые вопросы, не указывая автора этих слов?

 Да

 Нет

- 3. Ваш возраст?
- 4. Ваш пол?

 Женский

 Мужской

 Предпочитаю не отвечать

 Идентифицирую себя как:

- 5. Какое из следующих определений наиболее точно описывает вашу работу?

 Медсестра, ухаживающая за пациентами

 Врач, ухаживающий за пациентами

 Общинный медико-санитарный работник

 Другой поставщик медицинских услуг

 Руководитель медицинского учреждения или программы

 Исследователь

 Иное (уточните)

- 6. В организации какого типа вы работаете?

 Государственное учреждение здравоохранения

 **Частное, коммерческое** учреждение здравоохранения

 **Благотворительное/неприбыльное** учреждение здравоохранения

 Правительственная организация

 Местная неправительственная организация

 Международная неправительственная организация

 Финансирующая организация

 Университет или научное учреждение

 Иное (уточните)

- 7. Информацию о какой стране вы предоставляете?
- 8. Пожалуйста, укажите на вопросы какой категории вы будете отвечать (ТБ, ВИЧ либо обе категории)

 ТБ

 ВИЧ

 Обе категории

Пожалуйста, дайте ответ на 9 коротких вопросов о ТБ. Спасибо!

**Если вы желаете пропустить какие-то вопросы, выбирайте вариант «предпочитаю не отвечать».**

- 9. Стало ли **медицинским работникам сложнее добираться на работу** во фтизиатрические учреждения с началом COVID-19?

 Нет — все как раньше

 Да — немного сложнее

 Да — намного сложнее

 Да — очень сложно или невозможно

 Не знаю

 Предпочитаю не отвечать

- 10. Стало ли **пациентам с ТБ** сложнее **получать доступ к услугам в сфере ТБ** с началом COVID-19?

 Нет — все как раньше

 Да — немного сложнее

 Да — намного сложнее

 Да — очень сложно или невозможно

 Не знаю

 Предпочитаю не отвечать

- 11. Что вы считаете главными **проблемами или препятствиями для пациентов с ТБ** в контексте доступа к услугам здравоохранения с начала COVID-19? (выберите все подходящие пункты)

Социальное дистанцирование/карантинные правила

Нарушение работы транспорта

Снижение уровня доходов/доступа к средствам для поездок

Страх заразиться COVID-19

Закрытие лечебных учреждений

Дефицит поставщиков медицинских услуг

Длительное время ожидания

Дефицит масок

У пациентов с ТБ нет никаких проблем или препятствий

Предпочитаю не отвечать

Иное (уточните)

- 12. После начала COVID-19, какие **меры контроля внедрялись правительством** и как это повлияло на оказание услуг в сфере ТБ? (примеры: ограничение работы транспорта, ограничение передвижений и т.д.)

- 13. Известно ли вам о каких-либо изменениях **в работе фтизиатрических учреждений** после начала COVID-19? (выберите все подходящие пункты)

Нет — все как раньше

Да — протоколы социального дистанцирования для пациентов

Да — маски или иные средства защиты для медицинских работников

Предпочитаю не отвечать / не знаю

Да — Иное (уточните)

- 14. Сталкивались ли вы с дефицитом средств диагностики или иными проблемами в контексте оказания обычных **услуг диагностики** ТБ после начала COVID-19?

 Нет — все как раньше

 Да — стало немного сложнее предоставлять услуги диагностики

 Да — стало значительно сложнее предоставлять услуги диагностики

 Да — очень трудно или невозможно предоставлять услуги диагностики

 Не знаю

 Предпочитаю не отвечать

Ниже сообщите подробнее о том, что вызвало эти изменения

- 15. Сталкивались ли вы с дефицитом препаратов или иными проблемами в контексте предоставления стандартного **лечения** ТБ после начала COVID-19?

 Нет — все как раньше

 Да — стало немного сложнее предоставлять лечение ТБ

 Да — стало значительно сложнее предоставлять лечение ТБ

 Да — очень трудно или невозможно предоставлять лечение ТБ

 Не знаю

 Предпочитаю не отвечать

Ниже сообщите подробности, в т.ч. о проблемах с АРТ для пациентов с ТБ

- 16. Стало ли пациентам с ТБ получать доступ к **немедицинской поддержке** (например, дополнительному питанию или консультациям) после начала COVID-19?

 Нет — все как раньше

 Да — немного сложнее

 Да — намного сложнее

 Да — очень сложно или невозможно

 Недоступно в моей стране, регионе или учреждении

 Не знаю

 Предпочитаю не отвечать

Приведите подробности ниже

17. Что, по вашему мнению, можно сделать (или что уже было сделано) **для минимизации или исключения негативного влияния COVID-19** на услуги в сфере ТБ?

Нажав **ДАЛЕЕ**, вы завершите прохождение этого опроса. Пожалуйста, сначала проверьте свои ответы. Благодарим за участие в этом опросе!

Пожалуйста, дайте ответ на 9 коротких вопросов о ВИЧ. Спасибо!

**Если вы желаете пропустить какие-то вопросы, выбирайте вариант «предпочитаю не отвечать».**

- 18. Стало ли **медицинским работникам сложнее добираться на работу** в лечебные учреждения, работающие с ВИЧ, с началом COVID-19?

 Нет — все как раньше

 Да — немного сложнее

Да — намного сложнее

Да — очень сложно или невозможно

Не знаю

Предпочитаю не отвечать

- 19. Стало ли **пациентам с ВИЧ** сложнее **получать доступ к услугам в сфере ВИЧ** с началом COVID-19?

Нет — все как раньше

Да — немного сложнее

Да — намного сложнее

Да — очень сложно или невозможно

Не знаю

Предпочитаю не отвечать

- 20. Что вы считаете главными **проблемами или препятствиями для пациентов с ВИЧ** в контексте доступа к услугам здравоохранения с начала COVID-19?

Социальное дистанцирование/карантинные правила

Нарушение работы транспорта

Снижение уровня доходов/доступа к средствам для поездок

Страх заразиться COVID-19

Закрытие лечебных учреждений

Дефицит поставщиков медицинских услуг

Длительное время ожидания

Дефицит масок

У пациентов с ВИЧ сейчас НЕТ никаких проблем или препятствий

Предпочитаю не отвечать

Иное (уточните)

- 21. После начала COVID-19, какие **меры контроля внедрялись правительством** и как это повлияло на оказание услуг в сфере ВИЧ? (примеры: ограничение работы транспорта, ограничение передвижений и т.д.)

- 22. Известно ли вам о каких-либо изменениях **в работе лечебных учреждений в сфере ВИЧ** после начала COVID-19? (выберите все подходящие пункты)

Нет — все как раньше

Да — протоколы социального дистанцирования для пациентов

Да — маски или иные средства защиты для медицинских работников

Предпочитаю не отвечать / не знаю

Да — Иное (уточните ниже)

- 23. Сталкивались ли вы с дефицитом средств диагностики или иными проблемами в контексте оказания обычных **услуг диагностики** ВИЧ после начала COVID-19?

Нет — все как раньше

Да — стало немного сложнее предоставлять услуги диагностики

Да — стало значительно сложнее предоставлять услуги диагностики

Да — очень трудно или невозможно предоставлять услуги диагностики

Не знаю

Предпочитаю не отвечать

Приведите подробности ниже:

- 24. Сталкивались ли вы с дефицитом препаратов или иными проблемами в контексте предоставления стандартного **лечения** ВИЧ после начала COVID-19?

Нет — все как раньше

Да — стало немного сложнее предоставлять лечение ВИЧ

Да — стало значительно сложнее предоставлять лечение ВИЧ

Да — очень трудно или невозможно предоставлять лечение ВИЧ

Не знаю

Предпочитаю не отвечать

Приведите подробности ниже:

- 25. Стало ли пациентам с ВИЧ получать доступ к **немедицинской поддержке** (например, дополнительному питанию или консультациям) после начала COVID-19?

Нет — все как раньше

Да — немного сложнее

Да — намного сложнее

Да — очень сложно или невозможно

Недоступно в моей стране, регионе или учреждении

Не знаю

Предпочитаю не отвечать

Приведите подробности ниже

26. Что, по вашему мнению, можно сделать (или что уже было сделано) **для минимизации или исключения негативного влияния COVID-19** на услуги в сфере ВИЧ?

Нажав **ДАЛЕЕ**, вы завершите прохождение этого опроса. Пожалуйста, сначала проверьте свои ответы. Благодарим за участие в этом опросе!

**Сначала будут заданы 9 коротких вопросов о ТБ, а затем — 9 коротких вопросов о ВИЧ.**

**Если вы желаете пропустить какие-то вопросы, выбирайте вариант «предпочитаю не отвечать».**

**Благодарим за ваше время!**

- 27. Стало ли **медицинским работникам сложнее добираться на работу** во фтизиатрические учреждения с началом COVID-19?

Нет — все как раньше

Да — немного сложнее

Да — намного сложнее

Да — очень сложно или невозможно

Не знаю

Предпочитаю не отвечать

- 28. Стало ли **пациентам с ТБ** сложнее **получать доступ к услугам в сфере ТБ** с началом COVID-19?

Нет — все как раньше

Да — немного сложнее

Да — намного сложнее

Да — очень сложно или невозможно

Не знаю

Предпочитаю не отвечать

- 29. Что вы считаете главными **проблемами или препятствиями для пациентов с ТБ** в контексте доступа к услугам здравоохранения с начала COVID-19? (выберите все подходящие пункты)

Социальное дистанцирование/карантинные правила

Нарушение работы транспорта

Снижение уровня доходов/доступа к средствам для поездок

Страх заразиться COVID-19

Закрытие лечебных учреждений

Дефицит поставщиков медицинских услуг

Длительное время ожидания

Дефицит масок

У пациентов с ТБ нет никаких проблем или препятствий

Предпочитаю не отвечать

Иное (уточните)

- 30. После начала COVID-19, какие **меры контроля внедрялись правительством** и как это повлияло на оказание услуг в сфере ТБ? (примеры: ограничение работы транспорта, ограничение передвижений и т.д.)

- 31. Известно ли вам о каких-либо изменениях **в работе фтизиатрических учреждений** после начала COVID-19? (выберите все подходящие пункты)

Нет — все как раньше

Да — протоколы социального дистанцирования для пациентов

Да — маски или иные средства защиты для медицинских работников

Предпочитаю не отвечать / не знаю

Да — Иное (уточните)

- 32. Сталкивались ли вы с дефицитом средств диагностики или иными проблемами в контексте оказания обычных **услуг диагностики** ТБ после начала COVID-19?

Нет — все как раньше

Да — стало немного сложнее предоставлять услуги диагностики

Да — стало значительно сложнее предоставлять услуги диагностики

Да — очень трудно или невозможно предоставлять услуги диагностики

Не знаю

Предпочитаю не отвечать

Ниже сообщите подробнее о том, что вызвало эти изменения:

- 33. Сталкивались ли вы с дефицитом препаратов или иными проблемами в контексте предоставления стандартного **лечения** ТБ после начала COVID-19?

Нет — все как раньше

Да — стало немного сложнее предоставлять лечение ТБ

Да — стало значительно сложнее предоставлять лечение ТБ

Да — очень трудно или невозможно предоставлять лечение ТБ

Не знаю

Предпочитаю не отвечать

Приведите подробности ниже

34. Стало ли пациентам с ТБ получать доступ к **немедицинской поддержке** (например, дополнительному питанию или консультациям) после начала COVID-19?

Нет — все как раньше

Да — немного сложнее

Да — намного сложнее

Да — очень сложно или невозможно

Недоступно в моей стране, регионе или учреждении

Не знаю

Предпочитаю не отвечать

Приведите подробности ниже

35. Что, по вашему мнению, можно сделать (или что уже было сделано) **для минимизации или исключения негативного влияния COVID-19** на услуги в сфере ТБ?

Пожалуйста, дайте ответ на 9 вопросов о ВИЧ

**Если вы желаете пропустить какие-то вопросы, выбирайте вариант «предпочитаю не отвечать».**

- 36. Стало ли **медицинским работникам сложнее добираться на работу** в лечебные учреждения, работающие с ВИЧ, с началом COVID-19?

Нет — все как раньше

Да — немного сложнее

Да — намного сложнее

Да — очень сложно или невозможно

Не знаю

Предпочитаю не отвечать

37. Стало ли **пациентам с ВИЧ** сложнее **получать доступ к услугам в сфере ВИЧ** с началом COVID-19?

Нет — все как раньше

Да — немного сложнее

Да — намного сложнее

Да — очень сложно или невозможно

Не знаю

Предпочитаю не отвечать

- 38. Что вы считаете главными **проблемами или препятствиями для пациентов с ВИЧ** в контексте доступа к услугам здравоохранения с начала COVID-19?

Социальное дистанцирование/карантинные правила

Нарушение работы транспорта

Снижение уровня доходов/доступа к средствам для поездок

Страх заразиться COVID-19

Закрытие лечебных учреждений

Дефицит поставщиков медицинских услуг

Длительное время ожидания

Дефицит масок

У пациентов с ТБ нет никаких проблем или препятствий

Предпочитаю не отвечать / не знаю

Иное (уточните)

- 39. После начала COVID-19, какие **меры контроля внедрялись правительством** и как это повлияло на оказание услуг в сфере ВИЧ? (примеры: ограничение работы транспорта, ограничение передвижений и т.д.)

- 40. Известно ли вам о каких-либо изменениях **в работе лечебных учреждений в сфере ВИЧ** после начала COVID-19? (выберите все подходящие пункты)

Нет — все как раньше

Да — протоколы социального дистанцирования для пациентов

Да — маски или иные средства защиты для медицинских работников

Предпочитаю не отвечать / не знаю

Да — Иное

- 41. Сталкивались ли вы с дефицитом средств диагностики или иными проблемами в контексте оказания обычных **услуг диагностики** ВИЧ после начала COVID-19?

Нет — все как раньше

Да — стало немного сложнее предоставлять услуги диагностики

Да — стало значительно сложнее предоставлять услуги диагностики

Да — очень трудно или невозможно предоставлять услуги диагностики

Не знаю

Предпочитаю не отвечать

Ниже сообщите подробнее о том, что вызвало эти изменения:

- 42. Сталкивались ли вы с дефицитом препаратов или иными проблемами в контексте предоставления стандартного **лечения** ВИЧ после начала COVID-19?

Нет — все как раньше

Да — стало немного сложнее предоставлять лечение ВИЧ

Да — стало значительно сложнее предоставлять лечение ВИЧ

Да — очень трудно или невозможно предоставлять лечение ВИЧ

Не знаю

Предпочитаю не отвечать

Приведите подробности ниже:

- 43. Стало ли пациентам с ВИЧ получать доступ к **немедицинской поддержке** (например, дополнительному питанию или консультациям) после начала COVID-19?

Нет — все как раньше

Да — немного сложнее

Да — намного сложнее

Да — очень сложно или невозможно

Недоступно в моей стране, регионе или учреждении

Не знаю

Предпочитаю не отвечать

Приведите подробности ниже

44. Что, по вашему мнению, можно сделать (или что уже было сделано) **для минимизации или исключения негативного влияния COVID-19** на услуги в сфере ВИЧ?

Нажав кнопку **ГОТОВО**, вы завершите прохождение этого опроса. Пожалуйста, сначала проверьте свои ответы. Благодарим за участие в этом опросе!
